# Age‐Dependent Metabolomic Signatures of Dietary Restriction in Mice

**DOI:** 10.1111/acel.70309

**Published:** 2025-12-01

**Authors:** Ji‐sue Lee, Vindya H. J. Hetti Arachchige, Eun‐Hee Kim, Eunjung Bang, Young‐Shick Hong

**Affiliations:** ^1^ Department of Biological Sciences Chonnam National University Gwangju Republic of Korea; ^2^ Division of Food and Nutrition Chonnam National University Gwangju Republic of Korea; ^3^ Center for Research Equipment Korea Basic Science Institute Cheongju‐si Chungbuk Republic of Korea; ^4^ Metropolitan Seoul Center Korea Basic Science Institute Seoul Republic of Korea

**Keywords:** aging, dietary restriction, longevity, metabolomics

## Abstract

Caloric (CR) or dietary (DR) restriction improves health and extends lifespan in multiple species. However, the beneficial effects of DR may diminish if introduced late in life, emphasizing the importance of timing for promoting healthspan and avoiding adverse outcomes. Using a metabolomics approach, we investigated the metabolic responses in plasma, liver, and kidney of mice on acute and chronic DR at various ages. Two hundred and five mice including young (2‐month‐old; *n* = 72), middle‐aged (6‐month‐old; *n* = 76), and old (17‐month‐old; *n* = 57) mice for DR were involved. No significant metabolic distinctions were observed during acute DR across different ages. Throughout chronic DR, hepatic glucose, glycogen, and glutathione levels—all of which decreased with age—were elevated in all mice, demonstrating an improvement in energy metabolism and enhanced protection against oxidative stress. We also found age‐dependent metabolic responses to DR. Specifically, in young mice, amino acids and lactate contributed to gluconeogenesis in the liver during chronic DR. In contrast, in middle‐aged and older mice, only fatty acids played a role in the energy supply within the liver. We noted significant hepatic glycogen accumulation in old mice, along with decreased levels of hepatic betaine and sarcosine in young mice, indicating the negative impact of chronic DR on liver function. The findings suggest that the most substantial benefits of DR occur in the middle stage of life, highlighting the need for tailored dietary intervention strategies to promote health span at different life stages.

## Graphical abstract

Elevated hepatic glutathione (GSH) and 3‐hydroxybutyrate (3‐HB) levels in all ages of mice demonstrate an enhanced protection against oxidative stress and improvement in energy metabolism during chronic dietary restriction (DR). However, high protein or muscle degradation in liver of young mice, and large accumulation of hepatic glycogen and excessive fatty acid oxidation in old mice highlight age‐dependent metabolic responses to chronic DR.

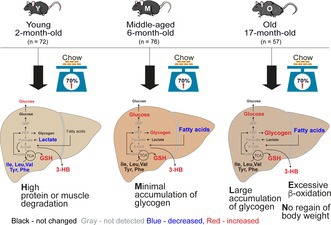

## 1 Introduction

Aging and aging‐related diseases have been widely introduced from molecular mechanisms to interventions and treatments (Guo et al. 2022). Healthy aging likely delays aging and reduces aging‐related impairments, thus extending lifespan. Many studies suggest that healthy aging is attributed to regular exercise, a healthy diet, regular doctor visits, and mental health care, even though genetics are not in our control. From a biological point of view, it is noteworthy that the most reliable, robust, and long‐known interventions for healthy aging target energy metabolism. For example, the attenuation of the growth hormone signaling induces the shift of energy resources from growth toward repair activities and thus extends lifespan (Brown‐Borg et al. 2014). Indeed, dietary restriction (DR) such as intermittent fasting (IF), time‐restricted feeding (TRF), and calorie restriction (CR) act by increasing metabolic plasticity. Metabolic plasticity is known as the metabolic resilience of the cells and their ability to swiftly alternate between metabolic substrates for energy production and decline with age (Chaudhari and Ermolaeva 2024; Longo and Anderson 2022). Therefore, since energy metabolism mainly occurs in mitochondria and aging significantly alters energy metabolism, modulation of mitochondrial resilience plays a crucial role in improving metabolic plasticity and thus extending lifespan. Chaudhari and Ermolaeva (2024) have demonstrated that the key variable for an effective metabolic intervention for healthy aging such as exercise and metformin, which elicit moderate mitochondrial and metabolic stress, as well as NAD+ boosters, rapamycin, and polymerase I inhibitors, which help reduce metabolic stress and molecular damage accumulation, is age. However, the effectiveness of these interventions may diminish over time due to the body's adaptive resilience and failure of mitochondrial function and plasticity mechanisms later in life. Therefore, these authors underline that the age‐adjustment concept of intervention for healthy aging may be an important step toward personalizing longevity treatments. Metabolic responses to DR were not associated with increased lifespan, indicating that improving health and extending lifespan are not synonymous and raising questions about which endpoints are the most relevant for evaluating aging interventions (Di Francesco et al. 2024). Indeed, mice subjected to 40% CR exhibited overall health benefits; however, they also experienced some adverse effects, including a lifelong loss of lean mass, lower body temperature, and alterations in their immune profile (Di Francesco et al. 2024). Therefore, it has been suggested that the health benefits of CR may vary significantly among individuals, depending on their genetic background (Di Francesco et al. 2024). Pak et al. (2021) have shed new light on how both when and how much we eat regulate metabolic health and longevity, and they demonstrated that daily prolonged fasting, following reduced caloric intake, is likely responsible for the metabolic and geroprotective benefits of the CR diet. Therefore, optimizing these benefits may require a tailored approach that considers factors such as age, the degree of CR, and the implementation of daily fasting alongside CR.

Metabolomics, the study of comprehensive small molecules in plants, microbial, animal and mammalian cells, tissues, and biofluids, is increasingly incorporated into aging research. With technological advancements with large datasets, metabolomics has enabled the population‐scale profiling of metabolites in humans, the findings of associations between multiple health conditions and metabolites, and thus prediction of aging, common incident diseases, and mortality risk (Cheng et al. 2015; Wang et al. 2011; Zhang et al. 2024). Taken together, age‐dependent metabolites in biofluids of humans and their associations with age‐related diseases have been widely explored (Lau et al. 2024; van den Akker et al. 2020; Yu et al. 2012). Over the last decade, a significant amount of metabolomic data has been collected, leading to the development of machine learning algorithms that create a metabolomic aging clock known as MileAge (Mutz et al. 2024). These algorithms can predict health and lifespan and are useful in health assessments, risk stratification, and proactive health monitoring, and thus promote healthy aging and longevity. In metabolomics studies on aging in animals (Houtkooper et al. 2011; Mitchell et al. 2016), CR is recognized as the most effective intervention for delaying aging and promoting healthy longevity. For example, metabolomics studies have revealed that CR‐induced Sirt3 regulates ornithine transcarbamoylase (OTC) activity and promotes the urea cycle and fatty acid oxidation, demonstrating improved mitochondrial function in the liver during CR (Hallows et al. 2011). Also, Aon et al. (2020) have found that fasting time and total calorie intake were determinants of increased survival in mice, rather than diet composition. DR is a broad term encompassing various feeding patterns such as CR, IF, TRF, specific nutrient restriction, and reduction in food intake. While CR focuses on an overall reduction of calories. these dietary patterns all retard the aging process, even though not in all mammals (Sohal and Forster 2014).

Di Francesco et al. (2024) noted that the effectiveness of CR may vary with age. While studies with dietary interventions aiming at promoting longevity are mostly applicable in young organisms, their impact on the metabolic health of older individuals under CR has not been thoroughly investigated. To address this gap, we conducted a study examining the effectiveness of DR in young, middle‐aged, and old mice through metabolomic analysis of their plasma, liver tissues, and kidney tissues. Based on previous findings that a classic 30% DR is the most effective dietary intervention for metabolic and geroprotective benefits (Pak et al. 2021), we chose a 30% DR diet for the mice in the current study. The mice were fed once a day and subjected to daily inter‐meal fasting with reduced food intake.

## 2 Results

**FIGURE 1:**
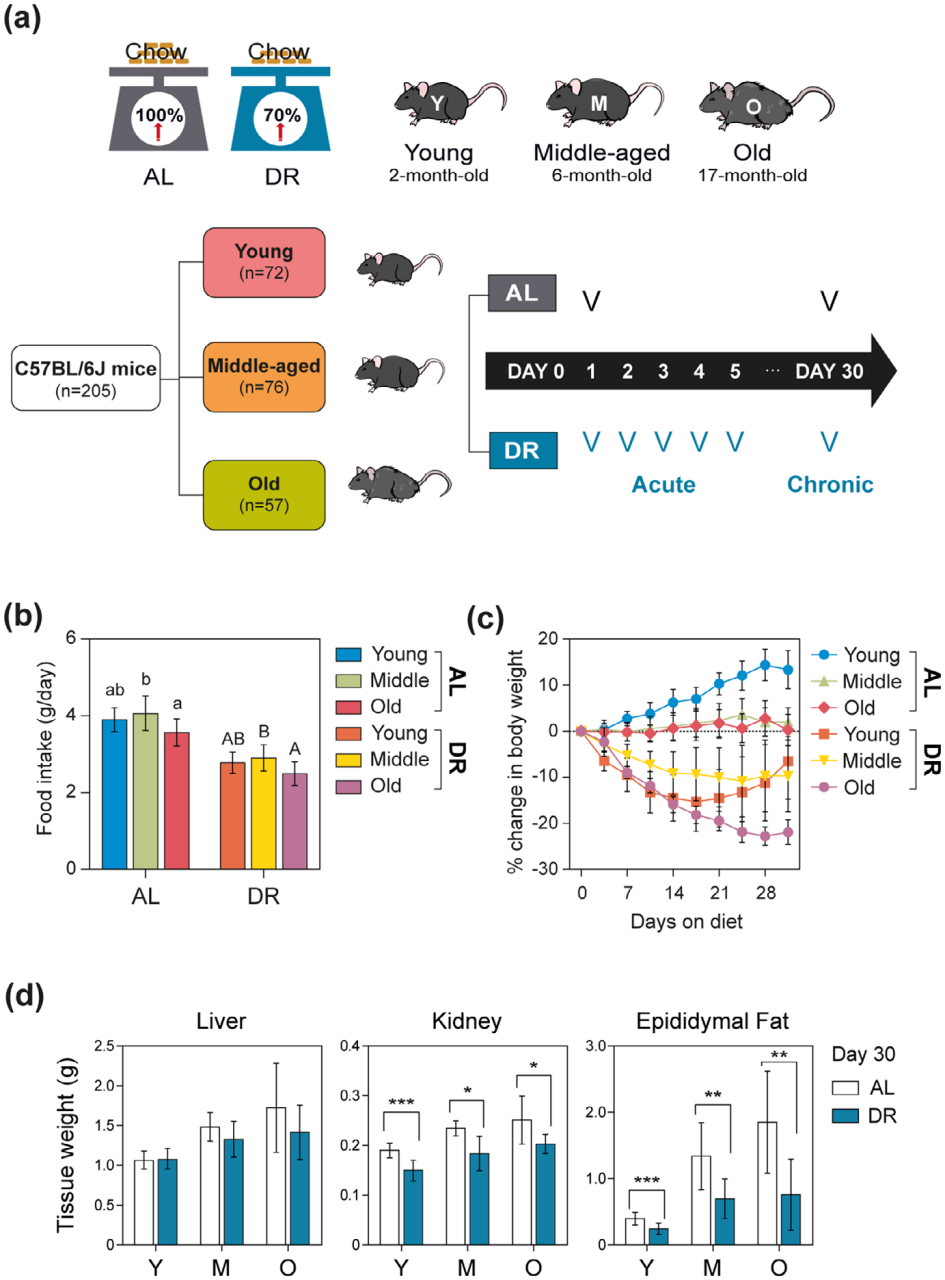
Dietary restriction (DR) causes different body weight and tissue changes in mice of different ages. (a) 30% DR at young (2‐month‐old, 5–6 weeks; *n* = 72), middle‐aged (6‐month‐old, 26–28 weeks; *n* = 76), and old (17‐month‐old, 73–75 weeks; *n* = 57) mice for acute DR for 5 days and chronic DR for 30 days. (b) Food intake of the ad libitum (AL) mice and DR mice during acclimation. (c) Changes in percent of body weight of all mice for chronic DR. (d) Relative weight (%) of liver, kidney, and epididymal fat after normalization to body weight of individual mouse. Data are presented as mean ± SD. Panel b was analyzed using one‐way ANOVA followed by Duncan's post hoc test to compare the differences among each of the three age groups in AL and DR. Panel (d) was analyzed using student's *t*‐tests to compare the AL with DR groups in the three age groups (*p < 0.05; **p < 0.01; ***p < 0.001).

**FIGURE 2:**
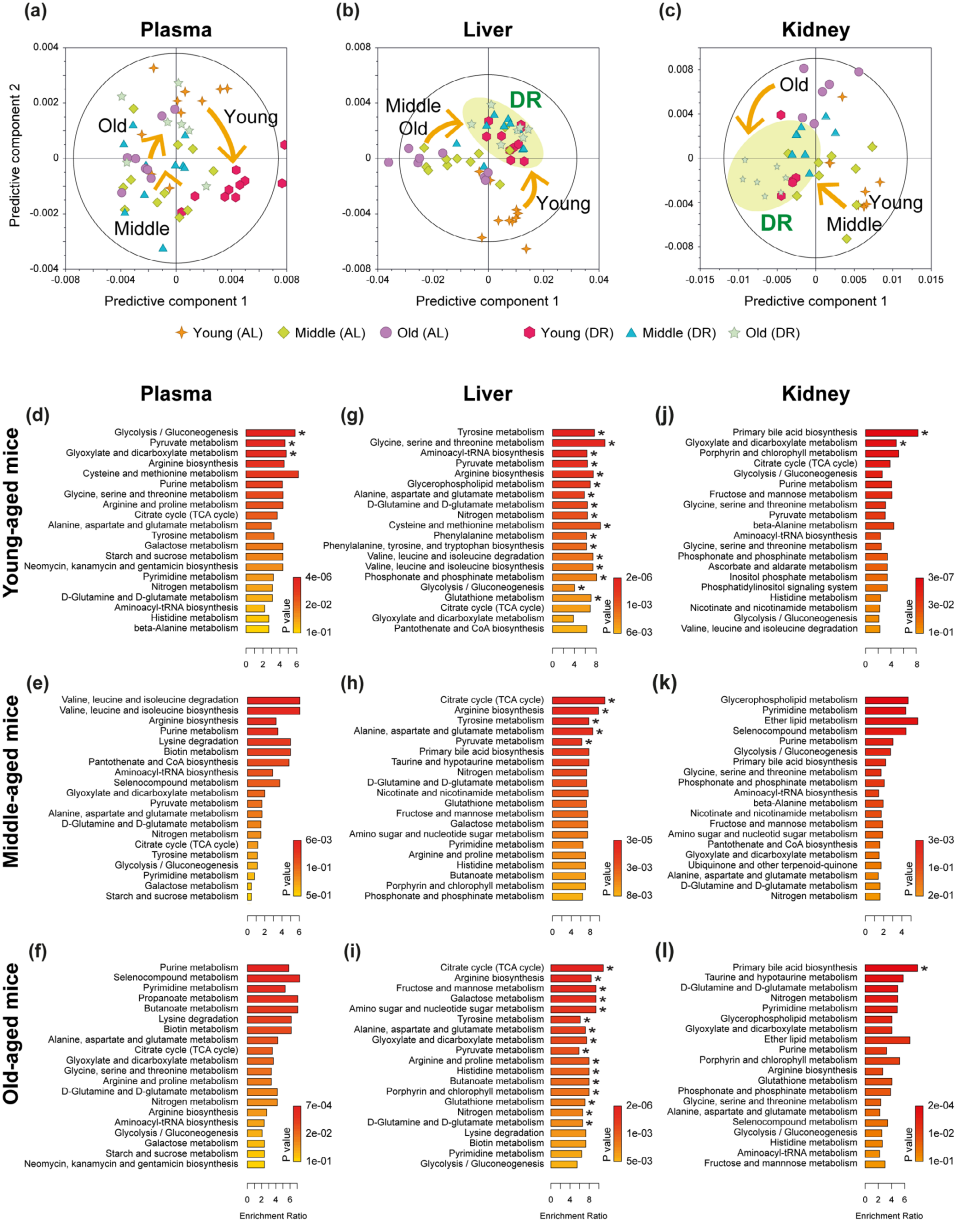
Metabolomics analysis reveals distinct metabolic pathways in plasma, liver, and kidney collected from mice of different ages during chronic DR. (a–c) OPLS‐DA score plots derived from general ^1^H NMR spectra of plasma and ^1^H MAS‐NMR spectra of liver and kidney. (d–i) Overview of metabolite set enrichment analysis (MSEA) based on a set of plasma (d–f), liver (g–i), and kidney (j–l) metabolites in DR young (d, g, and j), middle (e, h, and k), and old (f, i, and l) mice compared to AL mice. The metabolite sets that altered during DR are ranked according to their significance. Bar colors reflect the significance (raw *p*‐value) of the specific pathway. The length of each bar indicates the enrichment ratio. Metabolic pathways with asterisk indicate Holm *p*‐value < 0.05.

**FIGURE 3:**
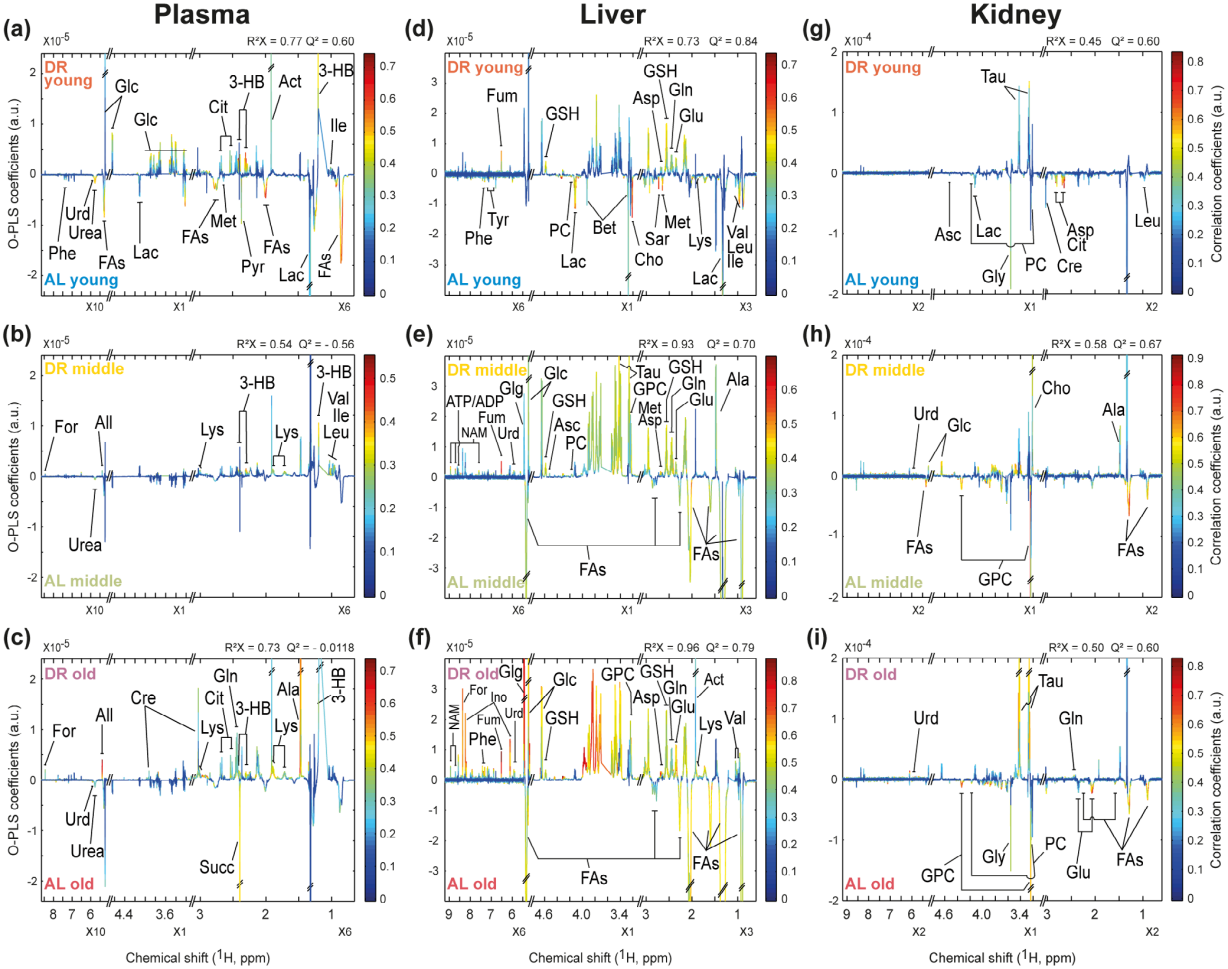
OPLS model‐based STOCSY analysis provides plasma, hepatic, and renal metabolites that were distinct between AL and DR mice during chronic DR in different‐aged mice. (a–c) STOCSY analysis derived from ^1^H NMR plasma spectra, showing pairwise comparisons of plasma metabolites. The plasma analyses of the AL young (*n* = 10), DR young (*n* = 10), AL middle‐aged (*n* = 11), DR middle‐aged (*n* = 10), AL old (*n* = 8), and DR old (*n* = 7) mice were conducted. (d–f) STOCSY analysis derived from ^1^H MAS‐NMR liver tissue spectra, showing pairwise comparisons of hepatic metabolites. The liver tissue analyses of the AL young (*n* = 10), DR young (*n* = 10), AL middle‐aged (*n* = 11), DR middle‐aged (*n* = 10), AL old (*n* = 8), and DR old (*n* = 7) mice are shown. (g–i) STOCSY analysis derived from ^1^H MAS‐NMR kidney tissue spectra, showing pairwise comparisons of renal metabolites. The kidney tissue analyses of the AL young (*n* = 9), DR young (*n* = 9), AL middle‐aged (*n* = 6), DR middle‐aged (*n* = 5), AL old (*n* = 8), and DR old (*n* = 7) mice are shown. 3‐HB, 3‐hydroxybutyrate; Ace, acetone; AcOH, acetate; Act, acetate; ADP, adenosine diphosphate; Ala, alanine; All, allantoin; Asc, ascorbate; Asp, aspartate; ATP, adenosine triphosphate; Bet, betaine; Cho, choline; Cit, citrate; Cre, creatine; For, formate; Fum, fumarate; Glc, glucose; Glg, glycogen; Gln, glutamine; Glu, glutamate; Gly, glycine; GPC, glycerophosphocholine; GSH, glutathione; Ile, isoleucine; Ino, inosine; Lac, lactate; Leu, leucine; Lys, lysine; Met, methionine; NAM, niacinamide; PC, phosphocholine; Phe, phenylalanine; Pyr, pyruvate; Sar, sarcosine; Succ, succinate; Tau, taurine; Tyr, tyrosine; Urd, uridine; Val, valine.

**FIGURE 4:**
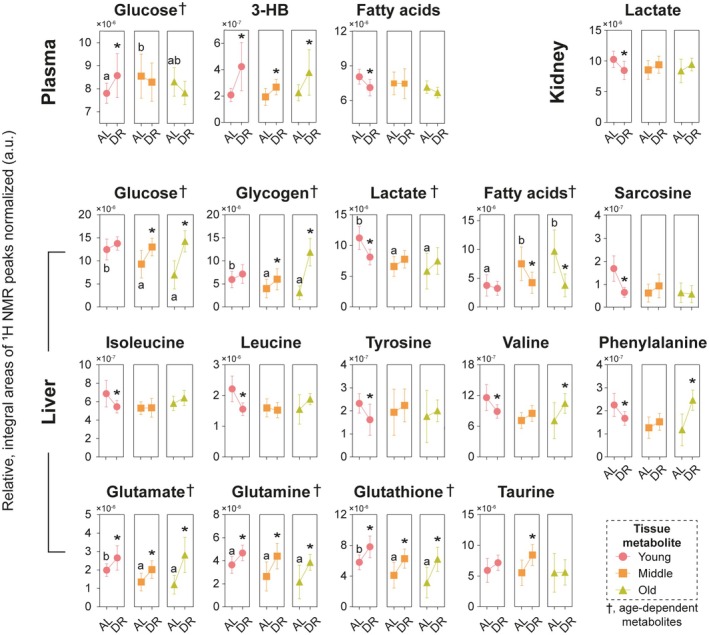
Perturbations in plasma, liver, and kidney tissue metabolites selected in young, middle‐aged, and old mice fed AL and with DR diets reflect their distinct metabolic responses to chronic DR. The symbols with pink, orange, and green represent the young, middle‐aged, and old mice, respectively. Data are presented as mean ± SD. Asterisks indicate significant differences in the levels of each tissue metabolite between AL and DR mice of the same ages. Different alphabets in metabolites marked with “†” denote significant differences in the corresponding metabolite levels among different aged mice fed AL. The statistical significance of the difference in the relative amounts of tissue metabolites was determined using unpaired t‐tests (*p < 0.05). 3‐HB, 3‐hydroxybutyrate.

To explore the dependences of the metabolic responses to DR on age, acute DR intervention for 5 days and chronic DR intervention for 30 days were applied to young (2‐month‐old, 5–6 weeks), middle‐aged (6‐month‐old, 26–28 weeks) and old (17‐month‐old, 73–75 weeks) mice (Figure 1a), in which control mice were fed ad libitum (AL) and 70% food was given for DR mice (30% DR). During both acute and chronic DR interventions, food intakes and body weight were monitored (Figure 1b,c), and ^1^H NMR‐based metabolomics approach was involved in plasma, liver, and kidney to investigate the metabolic effect of DR at different ages (Figures 2, 3, 4). The representative 1D ^1^H NMR spectra of plasma, liver, and kidney from control or AL young mice are shown in Figure S1. Metabolites identified in plasma, liver, and kidney included amino acid metabolism‐related metabolites (alanine, aspartate, betaine, creatine, glutamate, glutamine, glutathione, glycine, isoleucine, leucine, lysine, methionine, phenylalanine, sarcosine, tyrosine, valine, and taurine), glycolysis metabolism‐related metabolites (citrate, fumarate, glucose, glycogen, lactate, pyruvate, succinate, and myo‐inositol), energy status‐related metabolites (AMP, ATP/ADP, niacinamide, and 3‐hydroxybutyrate), and lipid metabolism‐related metabolites (fatty acids, betaine, choline, phosphorylcholine, and glycerophosphocholine).

### 2.1 Metabolic Effect of Acute DR

Compared with the mice before undergoing DR, the young mice experienced a loss of body weight like that of middle‐aged mice by Day 5 of DR. However, the most significant body weight loss was observed in the old mice (Figure S2a). We were able to identify the metabolites that changed in plasma, liver, and kidney every day during acute DR through pairwise comparisons between AL and DR mice for every day (1–5 days) and before DR (0 day) across young, middle‐aged, and old mice. The OPLS model‐based statistical TOCSY (STOCSY) analysis, generated from ^1^H NMR spectra of plasma, liver, and kidney collected during acute DR, was involved in the pairwise comparison, as illustrated in the STOCSY analysis (Figures S3–S11). Moreover, all metabolites from plasma, liver, and kidney were quantified by integral calculations of ^1^H NMR peaks corresponding to metabolites (Figures S12–S14).

In young mice of 2‐month‐old, the levels of fatty acids in plasma were increased at day 2 of DR until day 5, which were not observed in middle‐aged mice of 6‐month‐old and old mice of 17‐month‐old (Figures S3–S5 and S12). However, 3‐hydroxybutyrate, a typical ketone body, started increasing at day 4 of DR in the plasma of young mice, at day 2 of DR in the plasma of middle‐aged mice, and at day 1 of DR in the plasma of old mice. These results demonstrated the age dependence of DR on energy metabolism during acute DR. Unique changes in the hepatic metabolites were not observed in all ages of mice, following acute DR (Figures S6–S8). One notable change in the liver was the accumulation of glycogen in the young and middle‐aged mice during acute DR, whereas hepatic lipids tended to increase only in old mice during acute DR (Figure S13). Nevertheless, the levels of betaine in the liver of DR young mice were significantly decreased from Day 3 to Day 5, which was not observed in DR middle‐aged and old mice. Although statistical significance of renal metabolite changes in young and middle‐aged mice during acute DR was lacking, maybe due to the small number of mice used for analysis (Figures S9 and S10), the increase in levels of renal creatine in young mice at Days 4 and 5 of DR was clear (Figures S10 and S14). Furthermore, marked accumulations of lipids in the kidney of old mice during acute DR were found (Figures S11 and S14).

### 2.2 Metabolic Effects of Chronic DR

At Day 30 of the experiment, increased body weight was observed in AL young mice (Figure S2b), while body weights of AL middle‐aged and old mice remained unchanged. In contrast, significant weight losses were observed for all DR mice, as shown in Figure 1c. It was common to note that the changes in body weight reflect the active growth of young mice and the slower growth of middle‐aged and old mice when they eat food ad libitum. However, the effects of chronic DR on body weight varied by age throughout the 30 days of DR (Figures 1c and S2c). For instance, body weights of young mice increased after 14 days of DR, whereas body weights of middle‐aged mice remained stable. In contrast, old mice showed a continuous reduction in body weight by 30 days of DR. During chronic DR, liver weights increased only in young mice (Figures 1d and S2d), while no changes in kidney weights were observed across all age groups by Day 30 of DR. As expected, epididymal fat decreased in all mice during chronic DR, with the largest reduction in the old mice.

The OPLS‐DA score plots indicated metabolic changes in the plasma, liver, and kidney of all mice during chronic DR (Figure 2a–c). Notably, the hepatic metabolome distinguished AL young mice from AL middle‐aged and old mice; however, there was no clear differentiation between AL middle‐aged and old mice groups. These findings suggest that the dependence of hepatic metabolism on age during a normal diet diminishes after middle age, which is around 6 months in mice. Large metabolic changes in plasma during chronic DR were primarily observed in young mice, as indicated by the arrow in the OPLS‐DA score plot (Figure 2a). In contrast, substantial metabolic changes in liver and kidney tissues during chronic DR were evident across all age groups, from young to old mice.

In the metabolite set enrichment analysis (MSEA) to explore the metabolic pathway affected by chronic DR, the most significant changes in plasma metabolites were observed in young mice (Figure 2d–f). Notably, pathways related to glycolysis/gluconeogenesis, pyruvate metabolism, and glyoxylate/dicarboxylate metabolism were significantly altered in the plasma of DR young mice (Holm *p* < 0.05), which were less in DR middle‐aged and old mice. Furthermore, our analysis revealed that more metabolic pathways were significantly impacted in the liver by chronic DR compared to the plasma. Seventeen metabolic pathways were identified as altered in the liver of young mice, including tyrosine metabolism, glycine, serine, and threonine metabolism, aminoacyl‐tRNA biosynthesis, pyruvate metabolism, arginine biosynthesis, glycerophospholipid metabolism, alanine, aspartate, and glutamate metabolism, glutamine and glutamate metabolism, nitrogen metabolism, cysteine and methionine metabolism, phenylalanine metabolism, phenylalanine, tyrosine, and tryptophan biosynthesis, valine, leucine, and isoleucine degradation, valine, leucine, and isoleucine biosynthesis, phosphonate and phosphinate metabolism, glycolysis/gluconeogenesis, and glutathione metabolism. In the liver of middle‐aged mice, five metabolic pathways were affected, including citrate cycle, arginine biosynthesis, tyrosine metabolism, alanine, aspartate, and glutamate metabolism, and pyruvate metabolism. In the liver of old mice, 16 metabolic pathways were perturbed, including citrate cycle, arginine biosynthesis, fructose and mannose metabolism, galactose metabolism, amino sugar and nucleotide sugar metabolism, tyrosine metabolism, alanine, aspartate, and glutamate metabolism, glyoxylate and dicarboxylate metabolism, pyruvate metabolism, arginine and proline metabolism, histidine metabolism, butanoate metabolism, porphyrin and chlorophyll metabolism, glutathione metabolism, nitrogen metabolism, and glutamine and glutamate metabolism (Figure 2g–i). In the kidney of young mice, the MSEA suggested that metabolite changes were linked to primary bile acid biosynthesis and glyoxylate and dicarboxylate metabolism. In the kidney of old mice, only primary bile acid biosynthesis was indicated during chronic DR, with no statistically significant metabolic pathways identified in the kidneys of middle‐aged mice (Figure 2j–l).

### 2.3 Tissue‐Specific Metabolite Shifts During Chronic DR

To identify the individual metabolites perturbed in plasma, liver, and kidney during DR in mice across all groups, pairwise STOCSY analysis was produced from their whole ^1^H NMR datasets. Each OPLS model‐based STOCSY analysis was generated with one predictive and one orthogonal component, with their reliabilities and predictabilities described by R^2^X and Q^2^, respectively. In the STOCSY analysis, the upper section indicates higher levels of metabolites in the plasma of DR mice compared to those in the plasma of AL mice, while the lower section shows lower levels in the plasma of DR mice (Figure 3).

The STOCSY analysis also showed the most significant changes in plasma metabolites of young mice during chronic DR, with R^2^X = 0.77 and Q^2^ = 0.60. The STOCSY analysis demonstrated a consistent increase of 3‐hydroxybutyrate in the plasma of young, middle‐aged, and old mice during chronic DR. Additionally, increased levels of alanine, allantoin, formate, and lysine were observed in the plasma of all age groups during chronic DR. However, in young mice, there were notable increases in acetate, citrate, isoleucine, α‐glucose, and β‐glucose after the chronic DR. Conversely, these young mice also exhibited decreased levels of fatty acids, lactate, methionine, phenylalanine, pyruvate, and urea (Figure 3a–c).

During chronic DR, metabolic differentiations in the liver were observed between the AL and DR groups in young, middle‐aged, and old mice, in STOCSY analysis, as shown with good fitness, with R^2^X values of 0.73, 0.93, and 0.96, and high predictabilities indicated by Q^2^ values of 0.84, 0.70, and 0.79, respectively. The STOCSY analysis showed increased levels of aspartate, fumarate, glutamate, glutamine, and glutathione in the liver of DR young mice during chronic DR compared to AL young mice, along with decreased levels of betaine, choline, isoleucine, lactate, leucine, lysine, methionine, phenylalanine, phosphorylcholine, tyrosine, and valine in the liver of DR young mice during chronic DR (Figure 3d). The comparison of hepatic metabolites between the AL and DR groups in middle‐aged and old mice showed increased levels of aspartate, fumarate, glutamate, glutamine, glutathione, glycerophosphocholine, glycogen, niacinamide, phosphorylcholine, uridine, α‐glucose, and β‐glucose, following chronic DR (Figure 3e,f). In contrast, decreased levels of fatty acids were observed in the livers of middle‐aged and old mice after chronic DR.

The STOCSY analysis also exhibited that taurine levels were increased in the kidney tissues of DR young mice compared to AL young mice after chronic DR (Figure 3g). In contrast, the levels of ascorbate, aspartate, citrate, creatine, glycine, lactate, phosphorylcholine, and leucine were decreased in the kidney tissues of DR young mice. In the kidneys of DR middle‐aged mice, the amounts of alanine, choline, uridine, and glucose were found to be increased (Figure 3h), along with a decrease in fatty acids and glycerophosphocholine. For old DR mice, the levels of glutamine, taurine, and uridine were increased in kidney tissues during chronic DR compared to AL old mice (Figure 3i). Meanwhile, the levels of fatty acids, glutamine, glycerophosphocholine, glycine, and phosphorylcholine were decreased in the kidney tissues of DR old mice. Additionally, we provided all quantitative values of plasma, hepatic, and renal metabolites in DR mice compared to AL mice under chronic DR in Figures S15–S17,  Tables S1–S3 and Data S1.

## 3 Discussion

Aging is a natural process that occurs in all living organisms, but it increases susceptibility to various chronic diseases, physiological disorders, and cancer, which is considered a disease from certain perspectives. Therefore, modern society is increasingly focused on discovering breakthroughs to sustain health as we age, resulting in a wide range of interventions from lifestyle changes to therapeutic methods. Among these interventions, it is well established that DR or CR is the most effective strategy for delaying aging and extending lifespan. However, the benefits of DR can be limited if it is introduced later in life. The timing or age at which DR is initiated is crucial for realizing its benefits, as evidenced by nutritional memory effects observed in old mice (Hahn et al. 2019). In this study, we collected samples from the plasma, liver, and kidney of mice across three age groups: young, middle‐aged, and old. We examined metabolic alterations both during acute and chronic DR. The results revealed that metabolites in the plasma, liver, and kidney underwent distinct changes according to the age of mice both during a normal diet (AL) and DR intervention, demonstrating the complexity of metabolic adaptations at different life stages in mammals. During acute DR, we observed an increase in plasma fatty acid levels exclusively in young mice (Figure S12), indicating transient fatty acid mobilization from white adipose tissues (Selman et al. 2006). However, an increase in plasma 3‐hydroxybutyrate was observed in all age groups during acute DR, demonstrating an immediate energy requirement across ages.

The current study revealed not only metabolic changes during DR but also their dependence on age. Figure 4 presents the quantitative results for plasma, liver, and kidney metabolites that were found to be age‐dependent and uniquely altered during chronic DR. Previous research has widely reported age‐related variations in metabolites in plasma, muscle, and liver, highlighting their significant links to glucose and fat metabolism (Houtkooper et al. 2011). Our results confirmed that plasma glucose levels tend to increase with age (Figure 4), consistent with previous studies (Chen et al. 1985; Houtkooper et al. 2011; Reaven and Reaven 1980). Additionally, Pontzer et al. (2021) recently demonstrated that individuals over 60 experience reduced energy expenditure, as measured by total and basal energy levels across a large cohort ranging from 8 days to 95 years old. Therefore, it is common for healthy old mammals to show elevated plasma glucose levels, which may reflect a reduction in energy expenditure or a decline in glucose tolerance associated with aging.

In our study, we also observed that hepatic glucose levels gradually decreased with the age of mice fed AL (Figure 4). This finding contradicts results from a study on 23‐month‐old mice (Houtkooper et al. 2011), but aligns with results observed in 13‐month‐old mice (Atherton et al. 2009). Additionally, we noted reductions in hepatic glutathione levels and elevations in hepatic fatty acid levels with age, which are consistent with a previous report (Maher 2005). Consequently, the increases in plasma glucose and hepatic fatty acid levels, along with decreases in hepatic glucose, glycogen, lactate, and glutathione levels in aged AL‐fed mice, are likely related to declines in energy metabolism and antioxidant activity with age (Figure 4). These changes may serve as biomarkers for aging in our study.

**FIGURE 5:**
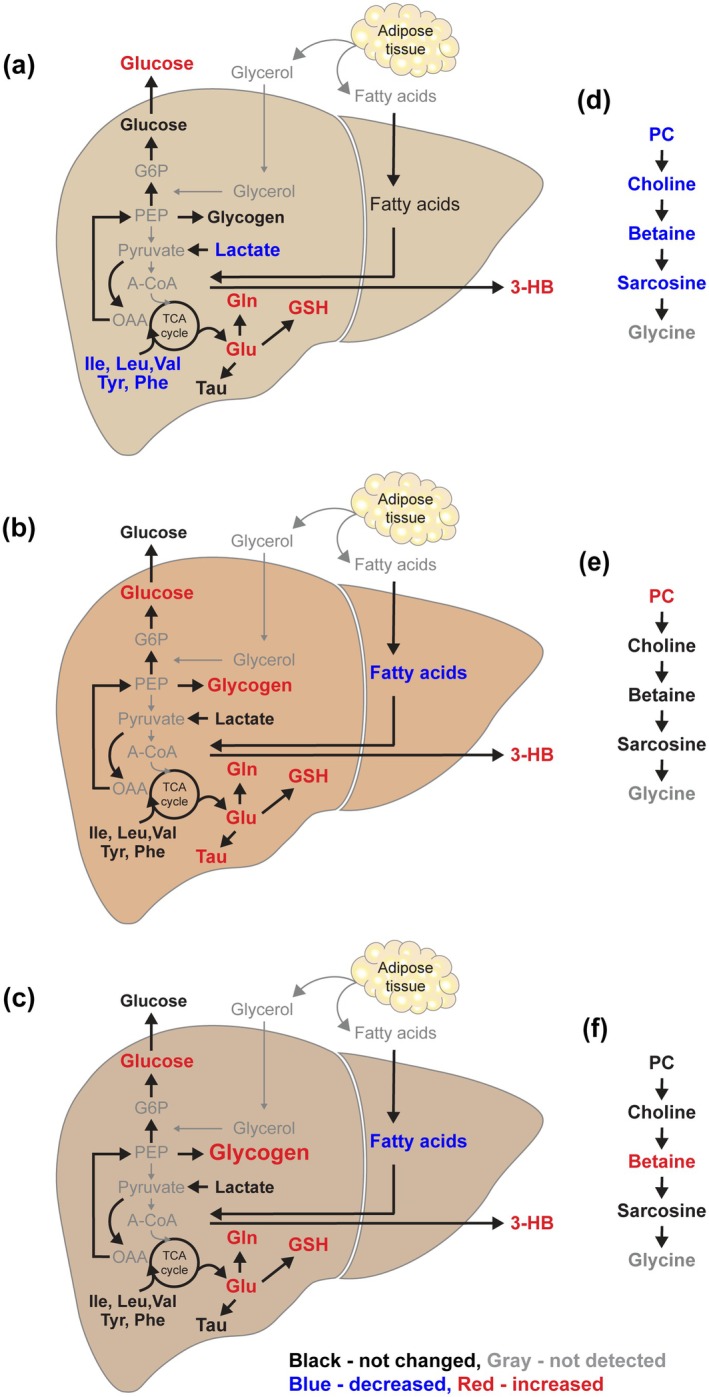
Age‐dependent metabolic responses to chronic DR in liver tissues include distinct gluconeogenesis, fatty acid oxidation, and glycogen accumulation. (a and d) Hepatic metabolites changed in the liver of young mice during chronic DR and their links to others. (b and e) Hepatic metabolites changed in the liver of middle‐aged mice during chronic DR and their links to others. (c and f) Hepatic metabolites changed in the liver of old mice during chronic DR and their links to others. 3‐HB, 3‐hydroxybutyrate; A‐CoA, acetyl‐coenzyme A; G6P, glucose 6‐phosphate; Gln, glutamine; Glu, glutamate; GSH, reduced glutathione; Ile, isoleucine; Leu, leucine; OAA, Oxaloacetate; PC, phosphorylcholine; PEP, phosphoenolpyruvate; Phe, phenylalanine; Tyr, tyrosine; Tau, taurine; Val, valine.

Elevations in the levels of plasma 3‐hydroxybutyrate were observed in all mice during acute and chronic DR, likely demonstrating energy demand or improved energy metabolism. Taken together, the findings of elevated glucose levels alongside reduced lactate, pyruvate, and fatty acid levels in plasma exhibited classical indicators of systemic gluconeogenesis. This systemic gluconeogenesis was observed only in young mice during chronic DR (Figure 5), consistent with results reported during fasting or DR (Kolb et al. 2021; Rui 2014). These results suggest that there are distinct systemic responses to DR between young and aged mice, reflecting different metabolic adaptations.

Due to energy deficiency during DR, increases in fasting blood glucose levels occur commonly, which likely result from gluconeogenesis (Rui 2014). Lactate, pyruvate, glycerol, and amino acids serve as substrates for gluconeogenesis mainly in the liver and kidney (Rui 2014). In the current study, increased fasting plasma glucose levels during chronic DR were observed only in young mice, alongside decreased levels of plasma pyruvate and lactate (Figures 3 and 4), highlighting a reflection of a systematic increase in gluconeogenesis among young mice during chronic DR. Additionally, local gluconeogenesis in the liver during chronic DR was evidenced by decreased hepatic lactate and glucogenic amino acid (alanine, isoleucine, valine, phenylalanine, and tyrosine) levels in young mice, even as hepatic glucose levels trended upward (Figure 5a).

In AL mice, hepatic glucose levels decreased with age (Figure 4), which is consistent with findings from previous studies (Houtkooper et al. 2011). Following chronic DR, hepatic glucose levels in middle‐aged and old mice increased to levels comparable to those in the young mice. However, there was no evidence of gluconeogenesis in the liver of middle‐aged and old mice during chronic DR, as indicated by the lack of significant changes in the levels of hepatic pyruvate, lactate, and amino acids, unlike in young mice. To date, glycerol, which is derived from triglycerides through lipolysis and serves as a gluconeogenic precursor, contributes to 22% of total glucose production during prolonged fasting in humans, such as after 62–86 h of starvation (Baba et al. 1995; Bortz et al. 1972; Landau et al. 1996; Shah and Wondisford 2020). Additionally, free fatty acids are well known to enhance hepatic gluconeogenesis through the production of acetyl‐CoA, NADH, and ATP, and by providing a source for gluconeogenesis in the glyoxylate and the xylulose 5‐phosphate pathways (Lam et al. 2003). Therefore, large reductions in hepatic fatty acid levels and marked elevations in hepatic glucose levels in both middle‐aged and old mice may reflect fatty acid‐attributed gluconeogenesis during chronic DR in the current study (Figure 5b,c). In fact, hepatic fatty acids accumulate with age due to reduced β‐oxidation of free fatty acids (Son et al. 2012). Only the increased fatty acid levels in aged mice were shared with obese mice, as reported (Serkova et al. 2006), consistent with the current study. This result demonstrates that other metabolites perturbed as mice aged were dependent on age in the current study, rather than obesity. These findings highlight that the gluconeogenesis observed in the liver during chronic DR differs between young and aged mice and contributes distinctly to glucose production on energy demand or energy deficiencies induced by a reduced diet. The liver provides 90%–95% of circulating glucose during the postabsorptive state (Chia et al. 2018). However, increases in hepatic glucose levels in middle‐aged and old mice during chronic DR did not affect their plasma glucose levels in the current study. This might be because hepatic glucose is involved in other metabolism during chronic DR, such as the accumulation of hepatic glycogen.

Glycogen breakdown in the liver, known as glycogenolysis, occurs during fasting to provide glucose as an energy source, leading to elevated blood glucose levels (Rui 2014). Glycogen can be directly observed in intact liver tissues by ^1^H MAS spectroscopy (Bollard et al. 2000). In the current study, marked accumulation of hepatic glycogen and increased hepatic glucose levels were observed in middle‐aged and old mice during chronic DR, with the effects being particularly pronounced in old mice (Figure 4). However, their blood glucose levels remained unchanged (Figure 4). Hepatic glycogen accumulation has rarely been reported in the condition of glucose deprivation or during dietary or calorie restriction. Nevertheless, Hu et al. (2022) demonstrated that active glycogen synthesis contributed to the elevated glycogen levels in the liver of mice undergoing a 40% calorie restriction (CR) to compensate for lipid insufficiency. Furthermore, hepatic glucose may have contributed to the accumulation of hepatic glycogen (excess glycogenesis), possibly because fatty acids were primarily used to compensate for energy deficiency during chronic DR (Galgani and Fernandez‐Verdejo 2021). This relationship was indicated by a strong inverse correlation between glucose and fatty acids in the liver tissues of middle‐aged (*p* < 0.001) and old (*p* = 0.008) mice in the current study (Figure S18). In contrast, the correlation observed in the liver of young mice was not significant (*p* = 0.117). Therefore, the increased glycogen levels in the livers of middle‐aged and old mice during chronic DR may reflect glycogenesis through the excess β‐oxidation of fatty acids in the liver (Figure 5b,c). Nevertheless, glycogen levels in the liver of middle‐aged mice subjected to chronic DR were found to be similar to those in AL young mice. The differences in glycogen levels between AL mice and chronic DR mice were measured at 1.85 ± 1.04, 1.80 ± 2.77, and 8.58 ± 3.55 for young, middle‐aged, and old mice, respectively. Also, the differences of hepatic fatty acids were −0.16 ± 1.04, −3.20 ± 3.96, and −5.45 ± 4.10 for young, middle‐aged, and old mice, respectively, after chronic DR when compared to AL mice. As a result, the large accumulation of hepatic glycogen in old mice during chronic DR suggests excess glycogenesis due to a corresponding increase in the breakdown of hepatic fatty acids. Furthermore, a study by de Toro‐Martin et al. (2020) indicated that early undernutrition leads to substantial hepatic glycogen accumulation in the liver and lack of glycogen mobilization after birth. These findings demonstrated increased glycogen synthesis and decreased endogenous glucose production in the livers of undernourished neonates, which is linked to reduced glucagon secretion [37]. Thus, large glycogen accumulation in the liver during food restriction may elevate the risk of developing metabolic diseases in the future.

Ketone bodies or ketones, produced in the body are used as an alternative energy source when glucose is scarce. In AL mice, plasma levels of 3‐hydroxybutyrate did not change with age. However, these levels increased in all DR mice during both acute and chronic DR (Figures 4 and S12), consistent with previous reports that ketone bodies are highly activated during CR or in conditions of glucose deprivation (Veech et al. 2017; Wijeyesekera et al. 2012). 3‐hydroxybutyrate, one of the most abundant ketone bodies in human circulation, is derived in the liver through the β‐oxidation of free fatty acids, which are produced by the lipolysis of adipose tissues during starvation, fasting, or when following a low carbohydrate diet (Veech et al. 2017). Mitochondrial β‐oxidation not only provides energy for hepatocytes but also generates ketone bodies. Therefore, ketone bodies inhibit gluconeogenesis in the liver, lipolysis in adipose tissue, glucose oxidation in the brain, and protein degradation in skeletal muscle (Fernandez‐Verdejo et al. 2023). In addition to CR, ketogenic diets also lead to an increase in circulating 3‐hydroxybutyrate, which contributes to extending lifespan (Roberts et al. 2017; Veech et al. 2017). It has also been reported that 3‐hydroxybutyrate supplementation can extend the lifespan of *C. elegans* (Edwards et al. 2014). Indeed, elevated levels of 3‐hydroxybutyrate have been shown to alleviate various age‐related diseases and improve health outcomes (Han et al. 2020). The increases in plasma 3‐hydroxybutyrate levels during DR indicate a shift from a state of energetic balance to one of energy deficit, which was observed in all mice during DR in the current study. This shift may contribute to the extension of a healthy lifespan. Specifically, increases in plasma 3‐hydroxybutyrate levels may reflect an increase in energy expenditure. Furthermore, fatty acids in the liver, which significantly increased with age, were found to be decreased in middle‐aged and old mice, suggesting a potential improvement in metabolic diseases. However, excess reductions of hepatic fatty acid levels in old mice during chronic DR could present issues.

Research has shown that as we age, glutathione levels decline in various tissues, including the liver. Glutathione is crucial for protecting against free radical damage associated with aging (Maher 2005). Several studies have reported that reduced glutathione (GSH) levels decreased in cells from old animals, while oxidized glutathione (GSSG) levels tend to increase (Sastre et al. 1996). In contrast to the findings of Houtkooper et al. (2011), hepatic glutathione levels in old mice were different in the current study (Figure 4). In comparison of serum metabolic differences among young (3–8 months), adult (13–23 months), and old (27–36 months) mice, serum levels of glucose, 3‐hydroxybutyrate, and glycerol were lower in the old mice [45]. However, the hepatic glutathione levels were found to be increased with age (Petr et al. 2021). Notably, discrepancies in the hepatic glutathione levels reported in previous studies compared to the current study may be attributed to the misidentification of reduced glutathione (GSH) as oxidized glutathione (GSSG), since GSH, an abundant form of glutathione, was not identified in the reports (Houtkooper et al. 2011; Petr et al. 2021). In our study, we clearly identified hepatic glutathione as GSH, and its levels were found to decrease with age. Notably, GSH levels were elevated in all mice during chronic DR (Figure 4), while their levels started to increase at Day 5 of acute DR in young and middle‐aged mice (Figure S13). Additionally, levels of hepatic glutamine and glutamate, which are precursors of glutathione, were positively associated with hepatic glutathione levels during chronic DR. These results indicate that chronic DR enhances protection against oxidative stress by removing reactive oxygen species through GSH.

Indeed, the markedly increased levels of taurine, an antioxidant in biological systems (Z. Y. Zhang et al. 2014), in the livers of only middle‐aged mice during chronic DR likely reflect an enhanced protective function of the liver in these middle‐aged mice (Figure 4). In young mice, hepatic taurine levels tended to increase after chronic DR, although this change was not statistically significant, and never changed in the old mice. Sarcosine is capable of inducing autophagy that helps cells survive, function, and protect liver cells under stress. However, sarcosine levels decline with age across multiple species (Walters et al. 2018). Glycine N‐methyltransferase (GNMT), which converts glycine to sarcosine, is predominantly expressed in the liver and its activity is suppressed as age increases (Johnson and Cuellar 2023). Walters et al. (2018) found that hepatic sarcosine levels remain relatively unchanged with aging but decrease with DR. These authors reported that plasma sarcosine levels decrease with age and increase with DR in both rodents and humans, which is attributed to liver GNMT activity. They also demonstrated that sarcosine could activate autophagy in cultured cells and enhance autophagic flux in vivo. Therefore, the marked reduction in hepatic sarcosine levels observed in young mice during chronic DR—bringing their levels down to those comparable to middle‐aged and old mice—may negatively impact liver functions (Figure 4). Young mice subjected to chronic DR may experience potential liver dysfunction due to decreased sarcosine levels. This reduction could be associated with liver malnutrition, resulting from gluconeogenesis, which might lead to excessive degradation of various amino acids. In contrast, middle‐aged mice demonstrate a tendency for increased hepatic sarcosine levels during chronic DR. This increase may support enhanced liver health and function in conjunction with elevated taurine levels in the liver.

Various dietary interventions have been introduced to extend lifespan, including dietary restriction, calorie restriction, time‐restricted feeding, and intermittent fasting. Among these dietary regimes, daily prolonged fasting combined with reduced caloric intake (CR‐DPF) has been found to be mostly effective for enhancing longevity and providing metabolic and geroprotective benefits. Additionally, it has been demonstrated that metabolic longevity treatments, such as DR and DR mimetic drugs, are mostly applicable in young and middle‐aged organisms, highlighting age limitations for these treatments. In the current study, young, middle‐aged, and old mice on a 30% DR received one meal a day at 7:00 p.m. and completed allotment within two hours. This resulted in an involuntary CR regime, leading to prolonged daily fasting until the next meal, similar to CR‐DPF. The metabolomic analysis of plasma, liver, and kidney samples from these mice revealed that chronic DR induced both systemic metabolite perturbations and gluconeogenesis in young mice, utilizing lactate and amino acids. In contrast, middle‐aged and old mice primarily relied on fatty acid oxidation for gluconeogenesis. Also, chronic DR may weaken liver function in young mammals possibly due to the high protein degradation for gluconeogenesis. In particular, the excessive oxidation of fatty acids and large glycogen accumulation in the livers of old mice during chronic DR, along with irreversible loss of body weight, highlight the potential harmful effects of chronic DR in aging mammals. Therefore, a personalized strategy for dietary or calorie intake reduction based on age groups, particularly for older mammals, is strongly recommended to avoid adverse effects.

## 4 Materials and Methods

### 4.1 Chemicals

Deuterium oxide (D_2_O, 99.9% D) containing 0.05 wt. % 3‐(trimethylsilyl)[2,2,3,3,‐D_4_] propionate (TSP) was purchased from Sigma (St. Louis, MO).

### 4.2 Animals

All experimental procedures were approved by the Institutional Animal Care and Use Committees at Chonnam National University (CNU‐IACUC‐YB‐2019‐68, Approved date: 03 September 2019). We obtained different ages C57BL/6J male mice (5–6, 26–28, and 73–75 weeks) from the Central Animal Laboratory (Daejeon, Korea) and the Korea Basic Science Institute (Daejeon, Korea). After arriving at our facilities, mice were housed individually (1 mouse per cage) with free access to food and drinking water. The animal facility was maintained at 22°C ± 2°C with 50% ± 10% humidity under a 12‐h light/dark cycle. Mice were fed a Harlen Teklad Global 18% Protein diet (Envigo, 2018S, Madison) and were acclimated for 2 weeks. During this acclimation period, individual food intake was monitored daily and determined. After the acclimation, mice were grouped randomly into an ad libitum group (AL) and a dietary restriction group (DR) in different age groups. And then, DR mice were fed a pellet adjusted to 70% of individual diet intake measurements at 10 a.m. every day for 30 days. While the DR group received a single meal of 70% of the food intake, the AL group was subjected to ad libitum access to food. On days 1,2,3,4,5, and 30, mice were sacrificed, and blood, liver, kidney, and fat tissue were collected. Blood samples collected in heparinized polythene tubes were centrifuged for 15 min at 1600 *g* and 4°C to obtain the plasma. All tissues were weighed and stored at −80°C until analysis.

### 4.3 Plasma Sample Preparation and ^1^H NMR Spectroscopic Analysis

An aliquot of 200 μL of plasma from each mouse was thawed and mixed with 400 μL of saline solution containing 30% D_2_O. Samples were centrifuged at 18214 *g* for 15 min at 10°C and then 550 μL supernatants were transferred into 5 mm NMR tubes for NMR analysis.

^1^H NMR spectra were acquired at 298 K on an Avance III700 spectrometer (Bruker BioSpin, Rheinstetten, Germany) equipped with a cryogenic triple‐resonance probe and a Bruker automatic injector, operating at a 700.40 MHz ^1^H frequency and using the water‐presaturated standard one‐dimensional Carr‐Purcell‐Meiboom‐Gill (1D CPMG) pulse sequence. Free induction decays (FID) of plasma were collected with 128 transients into 32 K data points using a spectral width of 7 kHz with a relaxation delay of 2.0 s, and an acquisition time of 2.123 s.

### 4.4 Liver and Kidney Sample Preparation and HR‐MAS ^1^H NMR Spectroscopic Analysis

Frozen tissue samples (8.5 ± 1 mg) were placed in a disposable insert tube. The tubes were filled with approximately 50 μL of D_2_O containing 0.05% TSP and inserted into a 4 mm ZrO2 rotor. All HR‐MAS ^1^H NMR spectra were acquired at 278 K on an Avance III700 spectrometer (Bruker BioSpin, Rheinstetten, Germany) equipped with a high‐resolution magic angle spinning (HR‐MAS) probe, operating at a 700.13 MHz ^1^H frequency and a spinning rate of 6 kHz. A 1D CPMG pulse sequence with water presaturation was applied for the acquisition of NMR spectra. For each sample, 128 transients were collected into 32 K data points using a spectral width of 14 kHz with a relaxation delay of 4.0 s, and an acquisition time of 1.162 s.

### 4.5 NMR Data Processing and Multivariate Statistical Analysis

All NMR spectra were manually adjusted using TOPSPIN (Version 4.1.4 Brucker Biospin, Rheinstetten, Germany) for phase and baseline distortions. After being transformed to American Standard Code for Information Interchange (ASCII) format, the spectra were calibrated to glucose (^1^H, 5.23 ppm) for plasma and alanine (^1^H, 1.48 ppm) for liver and kidney. Then, the spectra were aligned using the icoshift method in MATLAB (R2010b, Mathworks Inc., Natick, MA, USA) (Savorani et al. 2010). The regions corresponding to ethanol and water were removed prior to normalization and spectrum alignment. Normalization is a method of scaling each spectrum to the same virtual concentration in order to avoid the dilution effect of extracts and the effect of metabolites (Dieterle et al. 2006). After normalization, data were imported to SIMCA‐P version 17.0 (Umetrics, Umea, Sweden), and a mean‐centered scaling method was applied for multivariate statistical analysis. Orthogonal projection to latent structures discriminant analysis (OPLS‐DA), a supervised pattern recognition method was used to elicit maximum information about discriminant compounds from sample data (Bylesjö et al. 2006). Using MATLAB with scripts developed at Imperial College London (Cloarec et al. 2005), OPLS loading plots or OPLS model‐based statistical TOCSY (STOCSY) analysis results were generated with a color‐coded correlation coefficient for each piece of data to obtain highly interpretable models. The upper section in panels represents higher levels of metabolites in DR mice than in ad libitum mice, whereas the lower section represents lower levels of metabolites in DR mice. The color code in the loading plot corresponds to the correlation among variables. All OPLS‐DA models were generated with one predictive component and one orthogonal component. Model quality was assessed using the accumulative R^2^X and Q^2^ values, which indicate the reliability of fit and predictability, respectively.

### 4.6 Metabolite Enrichment Analysis

To highlight the most relevant altered pathways between AL and DR groups, Metabolite Set Enrichment Analysis (MSEA) was performed on the metabolites using the web‐based tool Metaboanalyst 5.0 (https://www.metaboanalyst.ca/) (Chong et al. 2018). The assignment of relative concentrations of metabolites was conducted by quantitative enrichment analysis based on Kyoto Encyclopedia of Genes and Genomes (KEGG) pathway. Metabolic pathways with a Holm *p*‐value, the *p*‐value adjusted by the Holm‐Bonferroni test, under 0.05 were considered significant.

### 4.7 Statistical Analysis

All experimental results were expressed as the mean ± SD. Student's *t*‐test was performed for unpaired samples to analyze the difference, and the significance of the differences was considered at *p* < 0.05. One‐way ANOVA was followed by Duncan's post hoc test to compare the differences among each of the three age groups in food intake monitoring. All statistical analyses were performed using SPSS Statistics version 26 (IBM Corporation, Armonk, NY, USA).

## Author Contributions

J.L. and Y.‐S.H. conceived and designed the study. J.L. and V.H.J.H.A. performed the experiments. J.L. and E.‐H.K. conducted the data analysis. J.L. and Y.‐S.H. wrote the manuscript. E.B. and Y.‐S.H. reviewed and revised the manuscript. All authors contributed to finalizing the manuscript.

## Funding

This study was supported by the National Research Foundation (NRF) grant funded by the Korea government (MIST) (RS‐2021‐NR059295).

## Conflicts of Interest

The authors declare no conflicts of interest.

## Supporting information


**FIGURE S1:** Representative 700 MHz 1H NMR and MAS‐NMR spectra of plasma, liver, and kidney tissues. (a) 700 MHz 1H NMR spectra of plasma. (b, c) 700 MHz 1H MAS‐NMR spectra of liver and kidney. The spectra were obtained from a young control mouse. Key: (1) VLDL/LDL; (2) fatty acids, (3) isoleucine; (4) leucine; (5) valine; (6) lactate; (7) alanine, (8) lysine, (9) acetate, (10) acetone, (11) 3‐hydroxybutyrate, (12) glutamate; (13) pyruvate; (14) succinate; (15) glutamine; (16) glutathione; (17) citrate; (18) methionine; (19) aspartate; (20) sarcosine; (21) cysteine; (22) creatine; (23) choline; (24) phosphorylcholine; (25) glycerophosphocholine; (26) taurine; (27) betaine; (28) myo‐inositol; (29) glycine; (30) ascorbate; (31) inosine; (32) beta‐glucose; (33) alpha‐glucose; (34) allantoin; (35) glycogen; (36) urea; (37) uridine; (38) adenosine tri/diphosphate; (39) adenosine monophosphate; (40) fumarate; (41) tyrosine; (42) histidine; (43) phenylalanine; (44) niacinamide; (45) formate.
**FIGURE S2:** Changes in body weight and tissue weight between AL and DR mice. (a) Percent changes in body weight of the acute DR group relative to the starting point for five days in all mice groups. (b) Comparison of body weight (g) between AL and chronic DR groups. (c) Changes in body weight (g) from the starting point for 30 days in all mice groups. (d) Tissue weight (g) of liver, kidney, and epididymal fat in all mice groups after 30 days. Data are presented as mean ± SD. Student's *t*‐tests were conducted to compare the AL and DR groups across the three age groups: paired *t*‐tests were used in panels (a) and (b), and unpaired *t*‐tests in panels (c) and (d) (*p < 0.05; **p < 0.01; ***p < 0.001).
**FIGURE S3:** Identifications of plasma metabolites changed in young mice during acute DR through STOCSY analysis. (a–e) STOCSY analyses that were derived from 1H NMR plasma spectra, showing pairwise comparisons of plasma metabolites. The plasma tissue analyses from young mice on day 0 (*n* = 9), day 1 (*n* = 9), day 2 (*n* = 8), day 3 (*n* = 8), day 4 (*n* = 8), and day 5 (*n* = 8) are shown. 3‐HB, 3‐hydroxybutyrate; All, allantoin; Cit, citrate; Fum, fumarate; Lac, lactate; Lys, lysine; Pyr, pyruvate; Succ, succinate; Tyr, tyrosine; Urd, uridine; Val, valine.
**FIGURE S4:** Identifications of plasma metabolites changed in middle‐aged mice during acute DR through STOCSY analysis. (a–e) STOCSY analyses that were derived from 1H NMR plasma spectra, showing pairwise comparisons of plasma metabolites. The plasma tissue analyses from middle mice on day 0 (*n* = 9), day 1 (*n* = 10), day 2 (*n* = 9), day 3 (*n* = 9), day 4 (*n* = 9), and day 5 (*n* = 9) are shown. 3‐HB, 3‐hydroxybutyrate; Cit, citrate; Glc, glucose; Lac, lactate; Lys, lysine; Met, methionine.
**FIGURE S5:** Identifications of plasma metabolites changed in old mice during acute DR through STOCSY analysis. (a–e) STOCSY analyses that were derived from 1H NMR plasma spectra, showing pairwise comparisons of plasma metabolites. The plasma tissue analyses from middle mice on day 0 (*n* = 7), day 1 (*n* = 7), day 2 (*n* = 7), day 3 (*n* = 7), day 4 (*n* = 7), and day 5 (*n* = 7) are shown. 3‐HB, 3‐hydroxybutyrate; Ala, alanine; Cit, citrate; Gln, glutamine; Succ, succinate; Urd, uridine.
**FIGURE S6:** Identifications of hepatic metabolites changed in young mice during acute DR through STOCSY analysis. (a–e) STOCSY analyses that were derived from 1H MAS‐NMR liver spectra, showing pairwise comparisons of hepatic metabolites. The liver tissue analyses from young mice on day 0 (*n* = 11), day 1 (*n* = 9), day 2 (*n* = 8), day 3 (*n* = 8), day 4 (*n* = 8), and day 5 (*n* = 8) are shown. ADP, adenosine diphosphate; ATP, adenosine triphosphate; Ala, alanine; Asc, ascorbate; Bet, betaine; Cho, choline; For, formate; Glc, glucose; Glg, glycogen; GSH, glutathione; Ino, inosine; Ile, isoleucine; Lac, lactate; Leu, leucine; Met, methionine; NAM, niacinamide; Phe, phenylalanine; pCho, phosphorylcholine; Sar, sarcosine; Tau, taurine; Urd, uridine; Val, valine.
**FIGURE S7:** Identifications of hepatic metabolites changed in middle‐aged mice during acute DR through STOCSY analysis. (a–e) STOCSY analyses that were derived from 1H MAS‐NMR liver spectra, showing pairwise comparisons of hepatic metabolites. The liver tissue analyses from middle mice on day 0 (*n* = 9), day 1 (*n* = 10), day 2 (*n* = 9), day 3 (*n* = 9), day 4 (*n* = 9), and day 5 (*n* = 9) are shown. Ace, acetone; ADP, adenosine diphosphate; ATP, adenosine triphosphate; Cho, choline; Glc, glucose; Gln, glutamine; Glg, glycogen; GSH, glutathione; NAM, niacinamide; Tau, taurine.
**FIGURE S8:** Identifications of hepatic metabolites changed in old mice during acute DR through STOCSY analysis. (a–e) STOCSY analyses that were derived from 1H MAS‐NMR liver spectra, showing pairwise comparisons of hepatic metabolites. The liver tissue analyses from old mice on day 0 (*n* = 7), day 1 (*n* = 7), day 2 (*n* = 7), day 3 (*n* = 7), day 4 (*n* = 7), and day 5 (*n* = 7) are shown. Ala, alanine; Cho, choline; Fum, fumarate; Ile, isoleucine; Val, valine.
**FIGURE S9:** Identifications of renal metabolites changed in young mice during acute DR through STOCSY analysis. (a–e) STOCSY analyses that were derived from 1H MAS‐NMR kidney spectra, showing pairwise comparisons of renal metabolites. The kidney tissue analyses from young mice on day 0 (*n* = 4), day 1 (*n* = 3), day 2 (*n* = 3), day 3 (*n* = 3), day 4 (*n* = 3), and day 5 (*n* = 3) are shown. Ala, alanine; Cre, creatine; Cys, cysteine; Glu, glutamate; Ino, inosine.
**FIGURE S10:** Identifications of renal metabolites changed in middle‐aged mice during acute DR through STOCSY analysis. (a–e) STOCSY analyses that were derived from 1H MAS‐NMR kidney spectra, showing pairwise comparisons of renal metabolites. The kidney tissue analyses from middle mice on day 0 (*n* = 4), day 1 (*n* = 5), day 2 (*n* = 4), day 3 (*n* = 4), day 4 (*n* = 4), and day 5 (*n* = 4) are shown. Ace, acetone; Ala, alanine; Asc, ascorbate; ADP, adenosine diphosphate; ATP, adenosine triphosphate; Cre, creatine; GPC, glycerophosphocholine; Gly, glycine; Ile, isoleucine; Ino, inosine; pCho, phosphorylcholine; Succ, succinate; Tyr, tyrosine; Val, valine.
**FIGURE S11:** Identifications of renal metabolites changed in old mice during acute DR through STOCSY analysis. (a–e) STOCSY analyses that were derived from 1H MAS‐NMR kidney spectra, showing pairwise comparisons of renal metabolites. The kidney tissue analyses from old mice on day 0 (*n* = 7), day 1 (*n* = 7), day 2 (*n* = 7), day 3 (*n* = 7), day 4 (*n* = 7), and day 5 (*n* = 7) are shown. Ace, acetone; Asc, ascorbate; Cit, citrate; Cho, choline; Cys, cysteine; Gln, glutamine; Leu, leucine; MI, myo‐inositol; pCho, phosphorylcholine; Tau, taurine; Urd, uridine.
**FIGURE S12:** Relative amounts of individual plasma metabolites during acute DR for 5 days in young, middle‐aged, and old mice. The symbols with pink, orange, and green colors represent the young, middle, and old mice groups, respectively. Data are presented as mean ± SD. Asterisks indicate significant differences in the levels of each plasma metabolite between AL at day 0 and DR mice of the same ages. The statistical significance of the difference in the relative amounts of plasma metabolites was determined using unpaired *t*‐tests (*p < 0.05).
**FIGURE S13:** Relative amounts of individual liver metabolites during acute DR of 5 days in young, middle‐aged, and old mice over a five‐day period. The symbols with pink, orange, and green colors represent the young, middle, and old mice groups, respectively. Data are presented as mean ± SD. Asterisks indicate significant differences in the levels of each liver metabolite between AL at day 0 and DR mice of the same ages. The statistical significance of the difference in the relative amounts of liver metabolites was determined using unpaired *t*‐tests (*p < 0.05).
**FIGURE S14:** Relative amounts of individual kidney metabolites during acute DR of 5 days in young, middle‐aged, and old mice over a five‐day period. The symbols with pink, orange, and green colors represent the young, middle, and old mice groups, respectively. Data are presented as mean ± SD. Asterisks indicate significant differences in the levels of each kidney metabolite between AL at day 0 and DR mice of the same ages. The statistical significance of the difference in the relative amounts of kidney metabolites was determined using unpaired *t*‐tests (*p < 0.05).
**FIGURE S15:** Relative amounts of individual plasma metabolites in young, middle‐aged, and old mice fed AL and with chronic DR for 30 days. The symbols with pink, orange, and green colors represent the young, middle‐aged, and old mice, respectively. Data are presented as mean ± SD. Asterisks indicate significant differences in the levels of each plasma metabolite between AL and DR mice of the same ages. The statistical significance of the difference in the relative amounts of plasma metabolites was determined using paired *t*‐tests (*p < 0.05). 3‐HB, 3‐hydroxybutyrate.
**FIGURE S16:** Relative amounts of individual hepatic metabolites in young, middle‐aged, and old mice fed AL and with chronic DR for 30 days. The symbols with pink, orange, and green colors represent the young, middle‐aged, and old mice, respectively. Data are presented as mean ± SD. Asterisks indicate significant differences in the levels of each liver metabolite between AL and DR mice of the same ages. The statistical significance of the difference in the relative amounts of liver metabolites was determined using paired *t*‐tests (*p < 0.05). GPC, glycerophosphocholine; pCho, phosphorylcholin.
**FIGURE S17:** Relative amounts of individual renal metabolites in young, middle‐aged, and old mice fed AL and with chronic DR for 30 days. The symbols with pink, orange, and green colors represent the young, middle‐aged, and old mice, respectively. Data are presented as mean ± SD. Asterisks indicate significant differences in the levels of each kidney metabolite between AL and DR mice of the same ages. The statistical significance of the difference in the relative amounts of kidney metabolites was determined using paired *t*‐tests (*p < 0.05). GPC, glycerophosphocholine; pCho, phosphorylcholin.
**FIGURE S18:** Pearson correlation between differences in fatty acids and glucose in the liver of mice during chronic DR. (a) The correlation between changes in hepatic fatty acid and glucose levels in young mice. (b) The correlation between changes in hepatic fatty acid and glucose levels in middle‐aged mice. (c) The correlation between changes in hepatic fatty acid and glucose levels in old mice.

**Table S1:** Quantitative results of plasma metabolites changed after DR in young, middle‐aged, and old mice.
**Table S2:** Quantitative results of hepatic metabolites changed after DR in young, middle‐aged, and old mice.
**Table S3:** Quantitative results of renal metabolites changed after DR in young, middle‐aged, and old mice.

**DataS1:** acel70309‐sup‐0003‐DataS1.xlsx.

## Data Availability

The authors have nothing to report.
