# Supplementary material for: Age‐Dependent Metabolomic Signatures of Dietary Restriction in Mice
Source: Aging Cell. 2025 Dec 1;25(1):e70309. doi: 10.1111/acel.70309 (PMC12740100; doi:10.1111/acel.70309)
Supplement: Supplementary file 1 — FIGURE S1: Representative 700 MHz 1H NMR and MAS‐NMR spectra of plasma, liver, and kidney tissues. (a) 700 MHz 1H NMR spectra of plasma. (b, c) 700 MHz 1H MAS‐NMR spectra of liver and kidney. The spectra were obtained from a young control mouse. Key: (1) VLDL/LDL; (2) fatty acids, (3) isoleucine; (4) leucine; (5) valine; (6) lactate; (7) alanine, (8) lysine, (9) acetate, (10) acetone, (11) 3‐hydroxybutyrate, (12) glutamate; (13) pyruvate; (14) succinate; (15) glutamine; (16) glutathione; (17) citrate; (18) methionine; (19) aspartate; (20) sarcosine; (21) cysteine; (22) creatine; (23) choline; (24) phosphorylcholine; (25) glycerophosphocholine; (26) taurine; (27) betaine; (28) myo‐inositol; (29) glycine; (30) ascorbate; (31) inosine; (32) beta‐glucose; (33) alpha‐glucose; (34) allantoin; (35) glycogen; (36) urea; (37) uridine; (38) adenosine tri/diphosphate; (39) adenosine monophosphate; (40) fumarate; (41) tyrosine; (42) histidine; (43) phenylalanine; (44) niacinamide; (45) formate. FIGURE S2: Changes in body weight and tissue weight between AL and DR mice. (a) Percent changes in body weight of the acute DR group relative to the starting point for five days in all mice groups. (b) Comparison of body weight (g) between AL and chronic DR groups. (c) Changes in body weight (g) from the starting point for 30 days in all mice groups. (d) Tissue weight (g) of liver, kidney, and epididymal fat in all mice groups after 30 days. Data are presented as mean ± SD. Student's t‐tests were conducted to compare the AL and DR groups across the three age groups: paired t‐tests were used in panels (a) and (b), and unpaired t‐tests in panels (c) and (d) (*p < 0.05; **p < 0.01; ***p < 0.001). FIGURE S3: Identifications of plasma metabolites changed in young mice during acute DR through STOCSY analysis. (a–e) STOCSY analyses that were derived from 1H NMR plasma spectra, showing pairwise comparisons of plasma metabolites. The plasma tissue analyses from young mice on day 0 (n = 9), day 1 [file ACEL-25-e70309-s003.docx]

**Supplementary Information**

**Age-Dependent Metabolomic Signatures of Dietary Restriction in Mice**

Ji-sue Lee^1^, Vindya H. J. Hetti Arachchige^2^, Eun-Hee Kim^3^, Eunjung Bang^4^, Young-Shick Hong^2^

^1^Department of Biological Sciences, Chonnam National University, Yongbong-ro, Buk-gu, Gwangju 61186, Republic of Korea

^2^Division of Food and Nutrition, Chonnam National University, Yongbong-ro, Buk-gu, Gwangju 61186, Republic of Korea

^3^Center for Research Equipment, Korea Basic Science Institute, Cheongwon‑Gu, Cheongju‑si, Chungbuk 28119, Republic of Korea

^4^Metropolitan Seoul Center, Korea Basic Science Institute, Seoul 03759, Republic of Korea


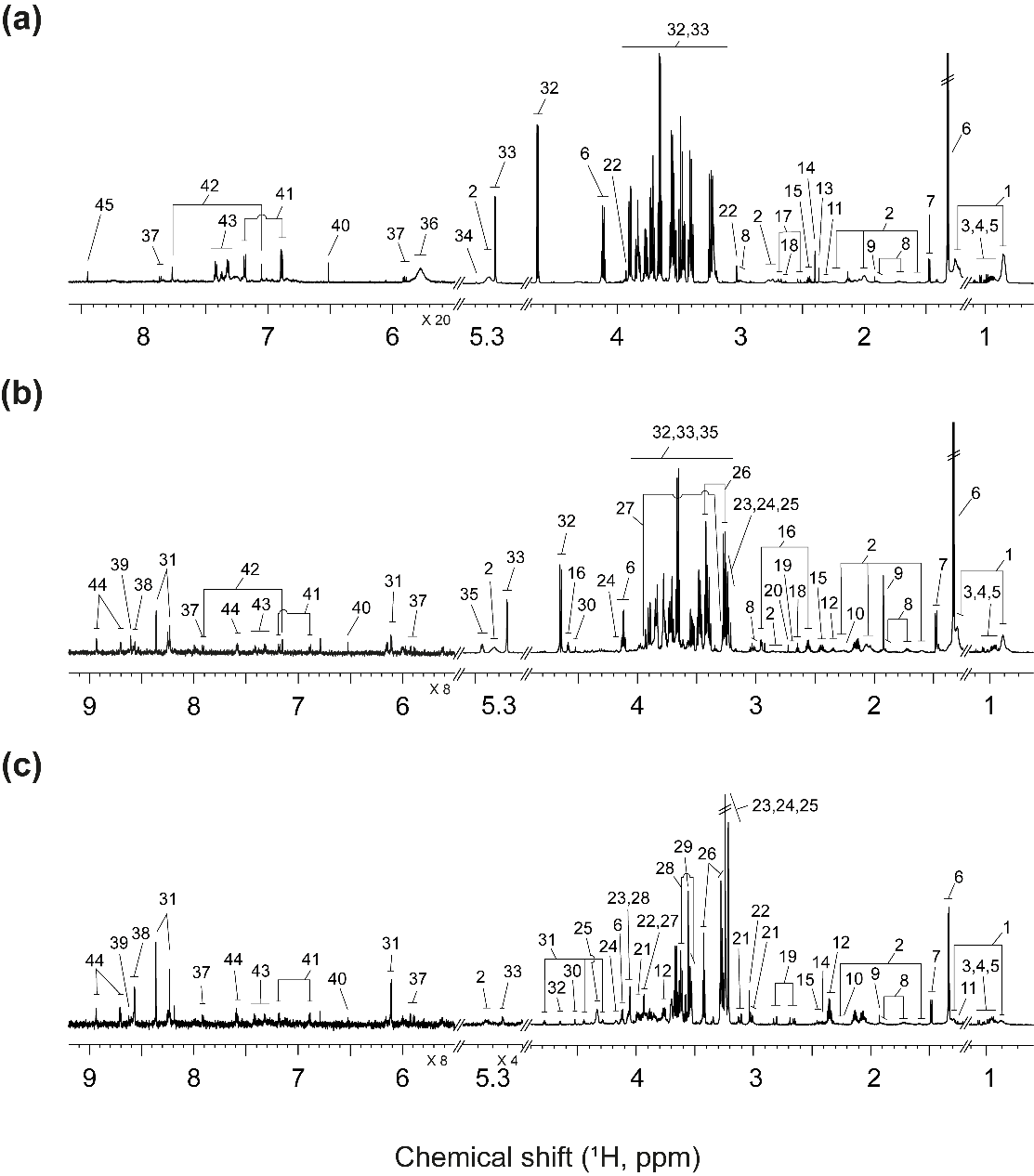


**FIGURE S1.** Representative 700 MHz ^1^H NMR and MAS-NMR spectra of plasma, liver, and kidney tissues. **(a)** 700 MHz ^1^H NMR spectra of plasma. **(b-c)** 700 MHz ^1^H MAS-NMR spectra of liver and kidney. The spectra were obtained from a young control mouse. Key: (1) VLDL/LDL; (2) fatty acids, (3) isoleucine; (4) leucine; (5) valine; (6) lactate; (7) alanine, (8) lysine, (9) acetate, (10) acetone, (11) 3-hydroxybutyrate, (12) glutamate; (13) pyruvate; (14) succinate; (15) glutamine; (16) glutathione; (17) citrate; (18) methionine; (19) aspartate; (20) sarcosine; (21) cysteine; (22) creatine; (23) choline; (24) phosphorylcholine; (25) glycerophosphocholine; (26) taurine; (27) betaine; (28) myo-inositol; (29) glycine; (30) ascorbate; (31) inosine; (32) beta-glucose; (33) alpha-glucose; (34) allantoin; (35) glycogen; (36) urea; (37) uridine; (38) adenosine tri/diphosphate; (39) adenosine monophosphate; (40) fumarate; (41) tyrosine; (42) histidine; (43) phenylalanine; (44) niacinamide; (45) formate

**
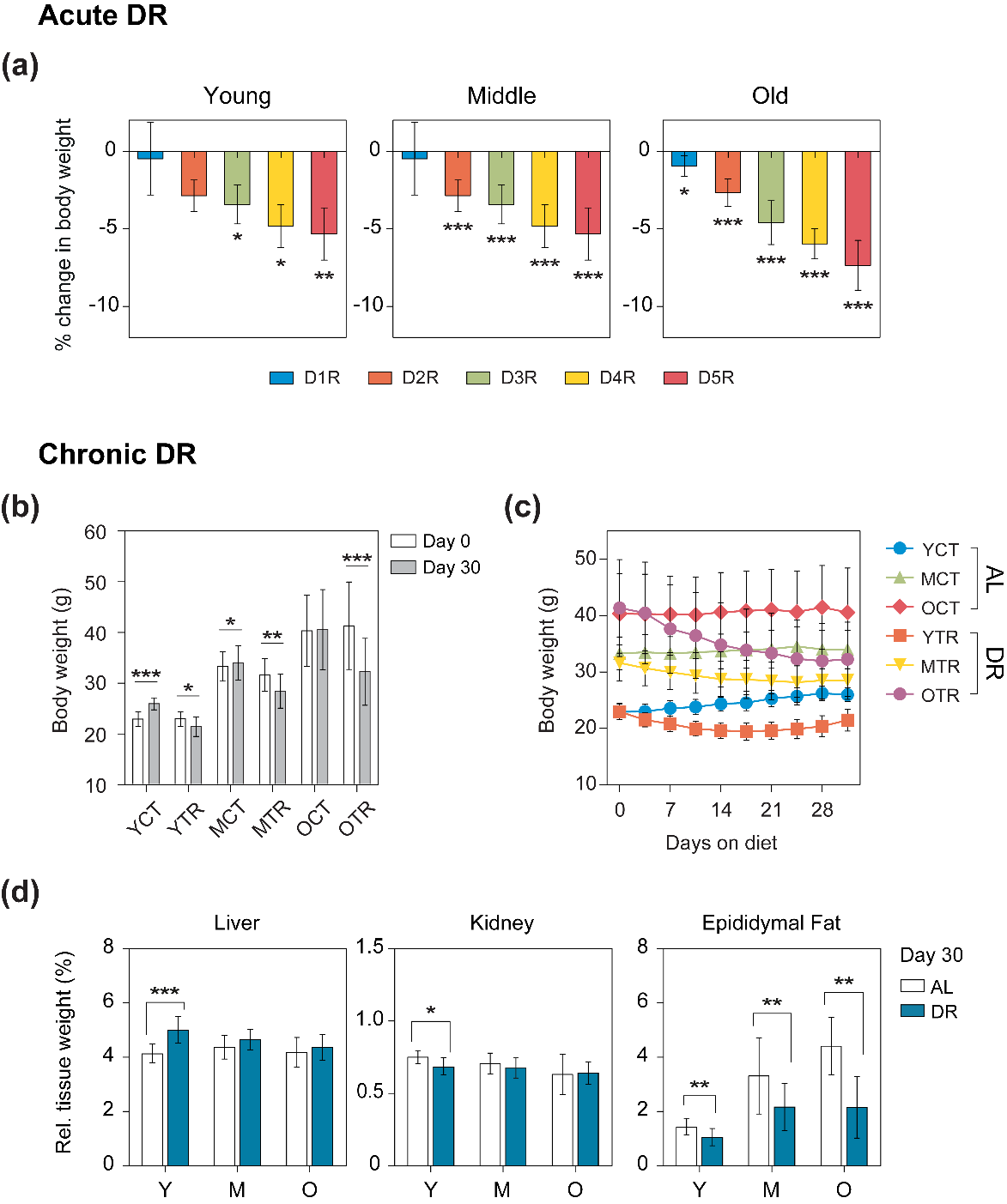
**

**FIGURE S2.** Changes in body weight and tissue weight between AL and DR mice. (a) Percent changes in body weight of the acute DR group relative to the starting point for five days in all mice groups. (b) Comparison of body weight (g) between AL and chronic DR groups. (c) Changes in body weight (g) from the starting point for 30 days in all mice groups. (d) Tissue weight (g) of liver, kidney, and epididymal fat in all mice groups after 30 days. Data are presented as mean ± SD. Student’s t-tests were conducted to compare the AL and DR groups across the three age groups: paired t-tests were used in panels (a) and (b), and unpaired t-tests in panels (c) and (d) (**p* < 0.05; ***p* < 0.01; ****p* < 0.001).


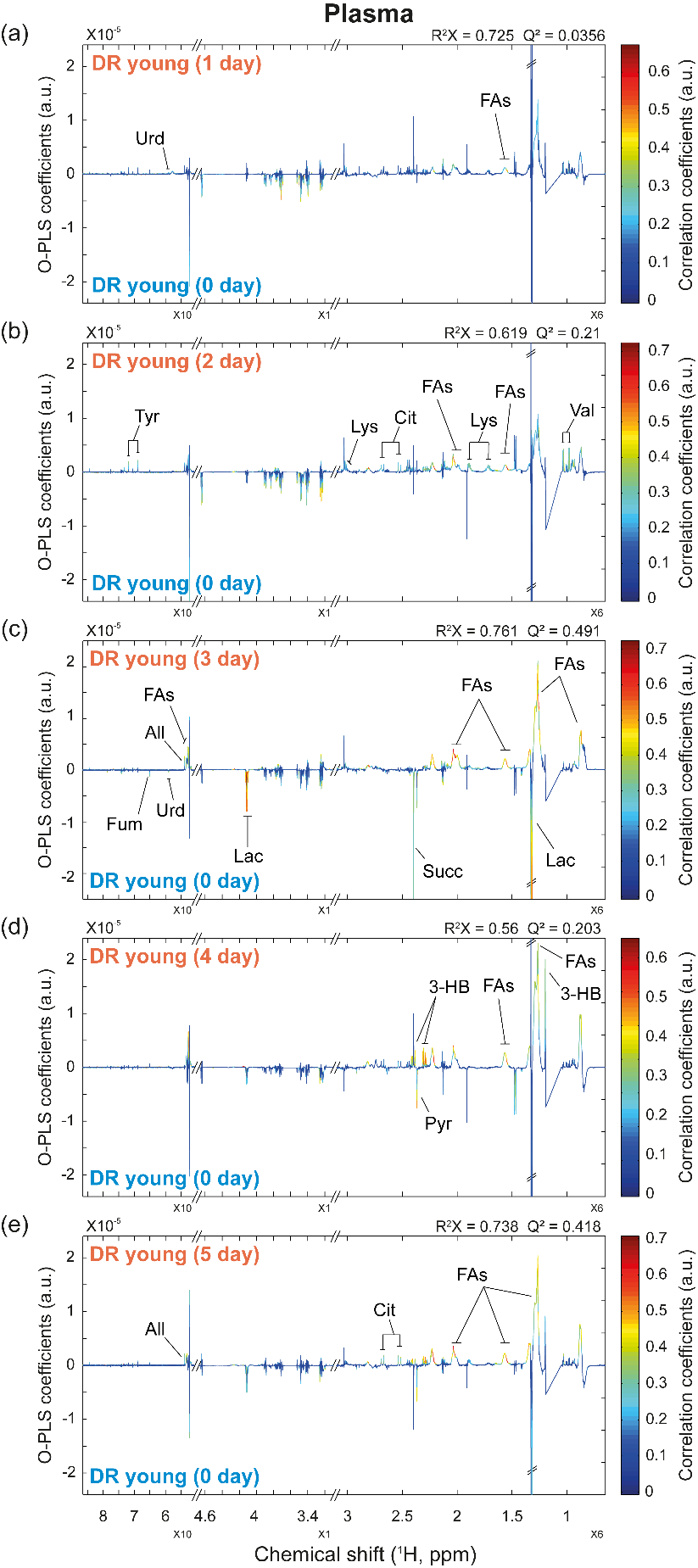


**FIGURE S3.** Identifications of plasma metabolites changed in young mice during acute DR through STOCSY analysis. (a-e) STOCSY analyses that were derived from ^1^H NMR plasma spectra, showing pairwise comparisons of plasma metabolites. The plasma tissue analyses from young mice on day 0 (*n* = 9), day 1 (*n* = 9), day 2 (*n* = 8), day 3 (*n* = 8), day 4 (*n* = 8), and day 5 (*n* = 8) are shown. 3-HB, 3-hydroxybutyrate; All, allantoin; Cit, citrate; Fum, fumarate; Lac, lactate; Lys, lysine; Pyr, pyruvate; Succ, succinate; Tyr, tyrosine; Urd, uridine; Val, valine.


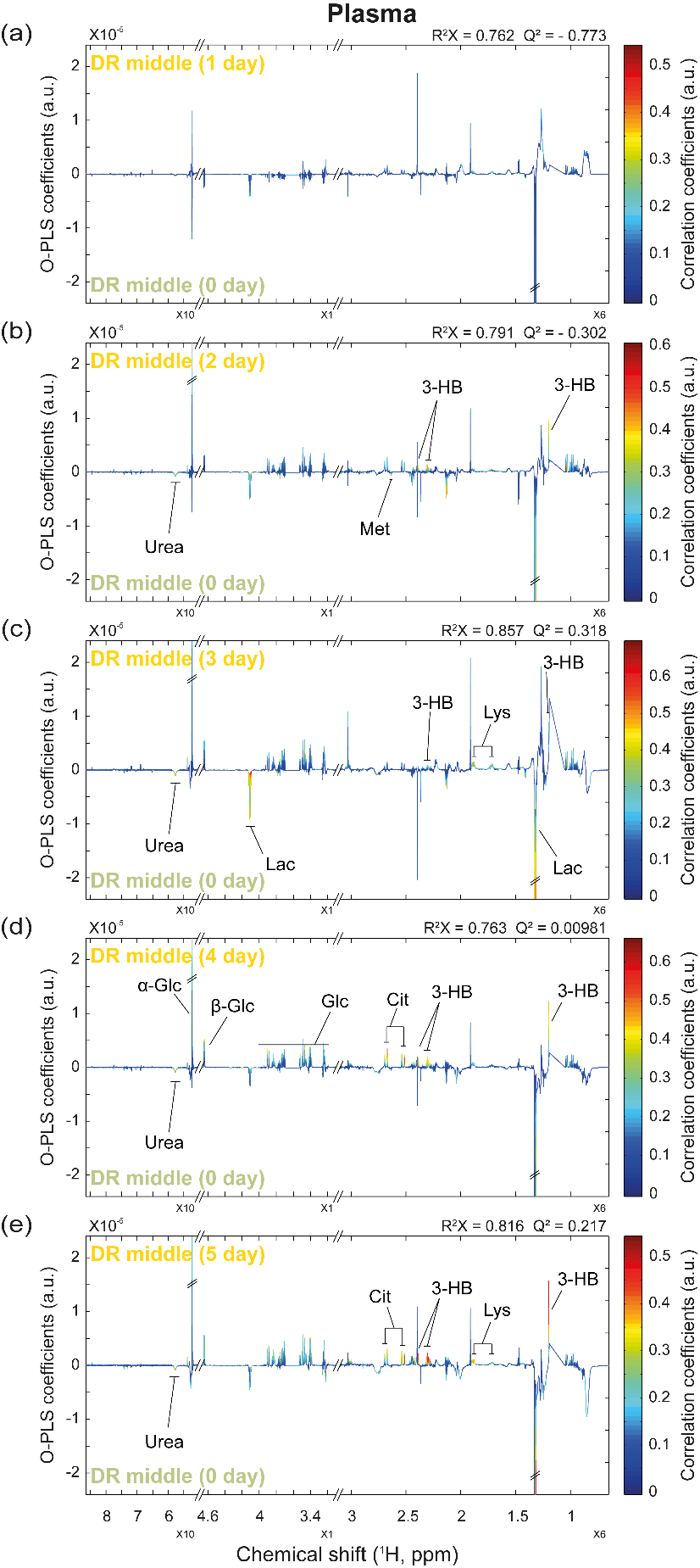


**FIGURE S4**. Identifications of plasma metabolites changed in middle-aged mice during acute DR through STOCSY analysis. **(a-e)** STOCSY analyses that were derived from ^1^H NMR plasma spectra, showing pairwise comparisons of plasma metabolites. The plasma tissue analyses from middle mice on day 0 (*n* = 9), day 1 (*n* = 10), day 2 (*n* = 9), day 3 (*n* = 9), day 4 (*n* = 9), and day 5 (*n* = 9) are shown. 3-HB, 3-hydroxybutyrate; Cit, citrate; Glc, glucose; Lac, lactate; Lys, lysine; Met, methionine.


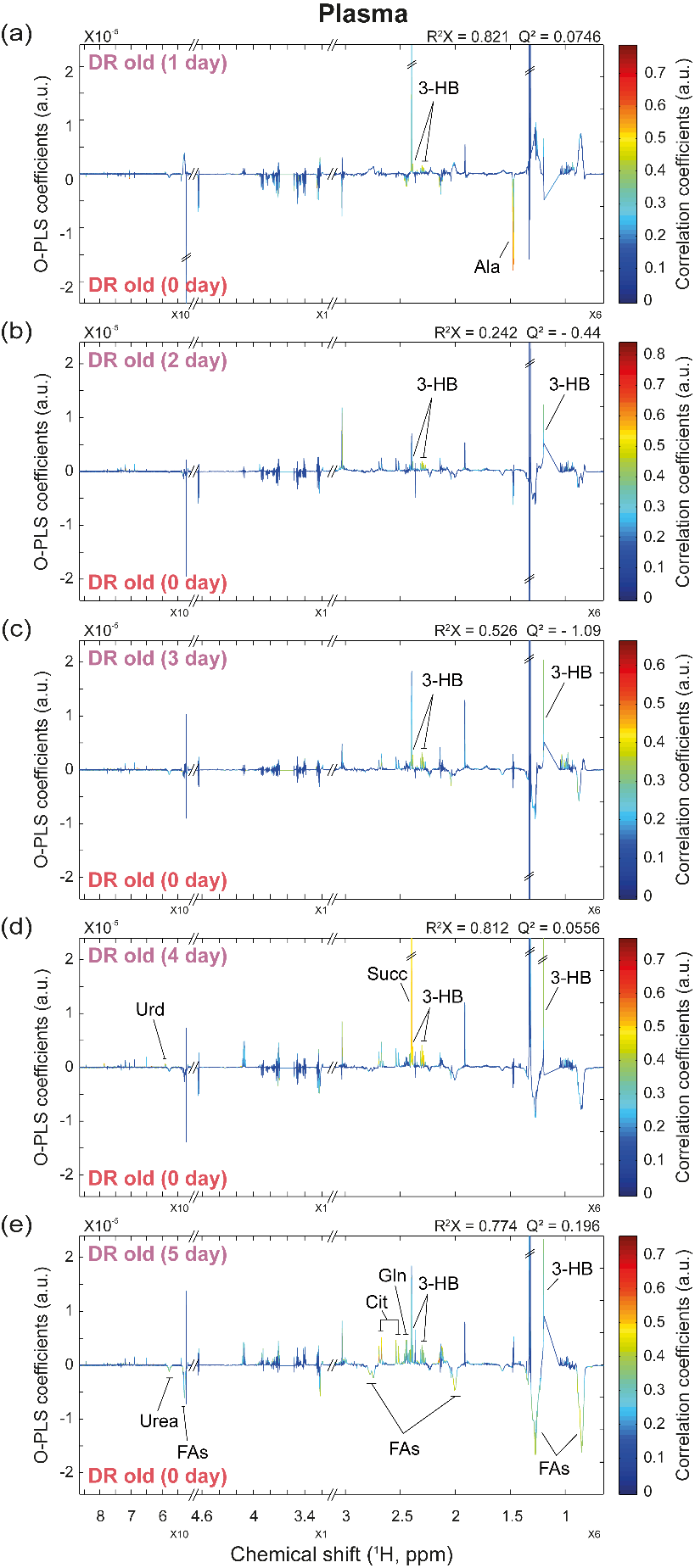


**FIGURE S5.** Identifications of plasma metabolites changed in old mice during acute DR through STOCSY analysis. **(a-e)** STOCSY analyses that were derived from ^1^H NMR plasma spectra, showing pairwise comparisons of plasma metabolites. The plasma tissue analyses from middle mice on day 0 (*n* = 7), day 1 (*n* = 7), day 2 (*n* = 7), day 3 (*n* = 7), day 4 (*n* = 7), and day 5 (*n* = 7) are shown. 3-HB, 3-hydroxybutyrate; Ala, alanine; Cit, citrate; Gln, glutamine; Succ, succinate; Urd, uridine.


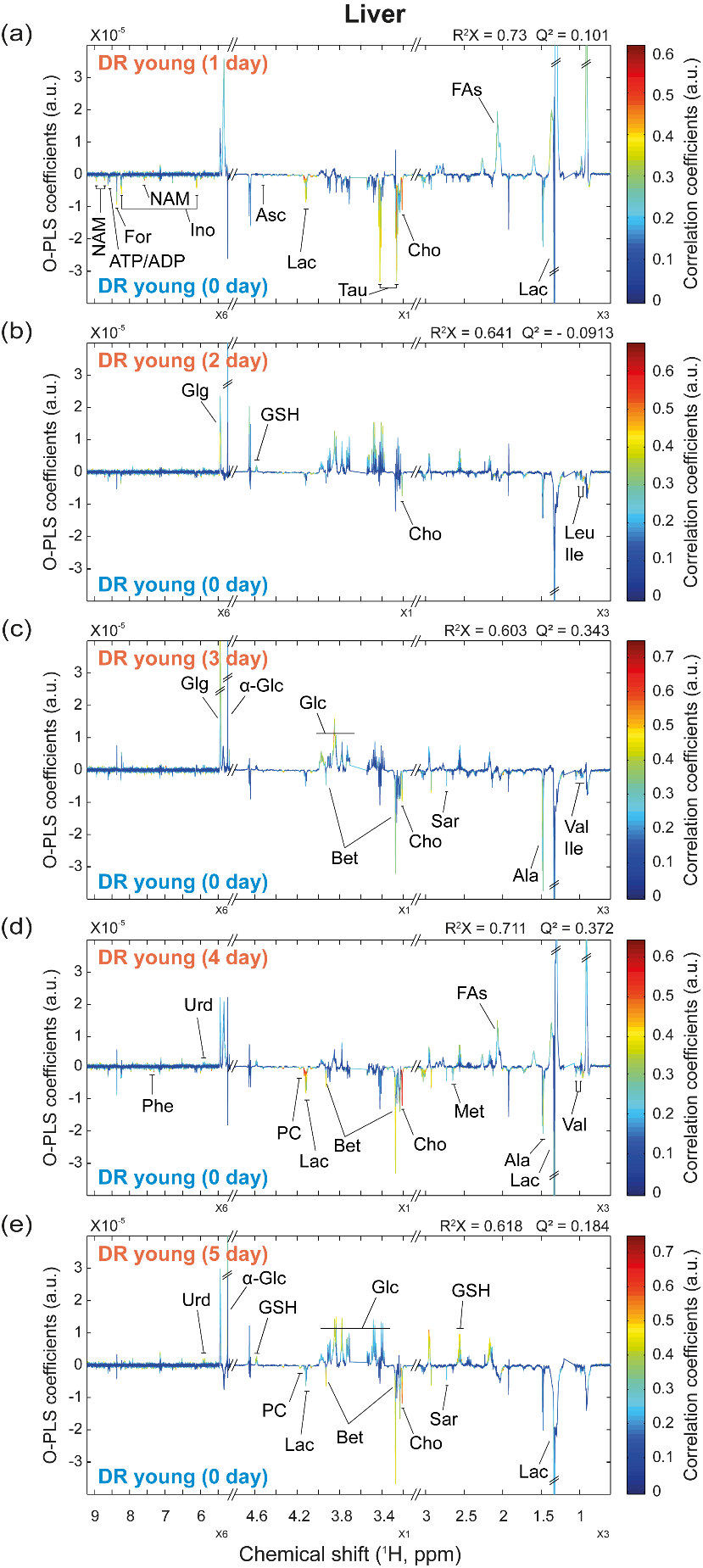


**FIGURE S6.** Identifications of hepatic metabolites changed in young mice during acute DR through STOCSY analysis. (a-e) STOCSY analyses that were derived from ^1^H MAS-NMR liver spectra, showing pairwise comparisons of hepatic metabolites. The liver tissue analyses from young mice on day 0 (*n* = 11), day 1 (*n* = 9), day 2 (*n* = 8), day 3 (*n* = 8), day 4 (*n* = 8), and day 5 (*n* = 8) are shown. ADP, adenosine diphosphate; ATP, adenosine triphosphate; Ala, alanine; Asc, ascorbate; Bet, betaine; Cho, choline; For, formate; Glc, glucose; Glg, glycogen; GSH, glutathione; Ino, inosine; Ile, isoleucine; Lac, lactate; Leu, leucine; Met, methionine; NAM, niacinamide; Phe, phenylalanine; PC, phosphorylcholine; Sar, sarcosine; Tau, taurine; Urd, uridine; Val, valine.


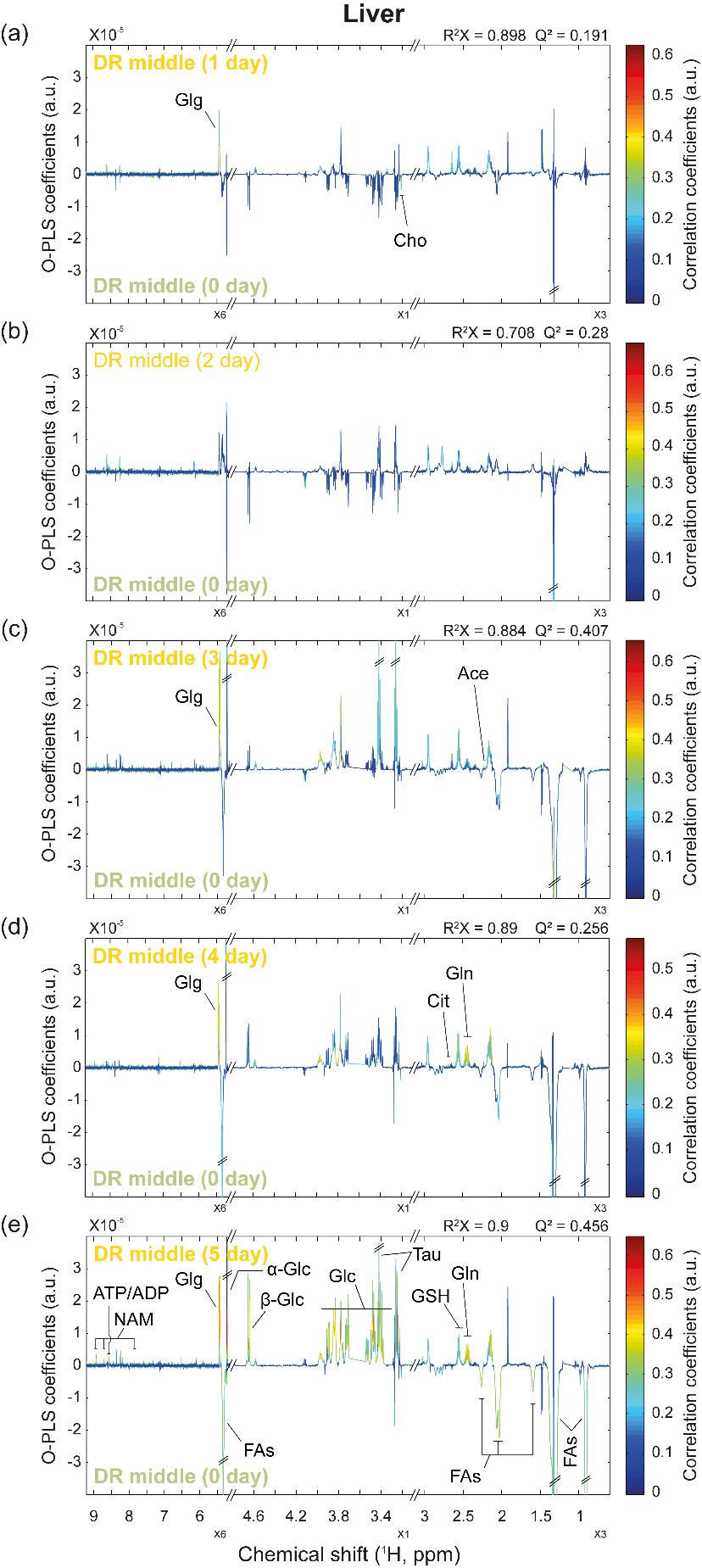


**FIGURE S7.** Identifications of hepatic metabolites changed in middle-aged mice during acute DR through STOCSY analysis. (a-e) STOCSY analyses that were derived from ^1^H MAS-NMR liver spectra, showing pairwise comparisons of hepatic metabolites. The liver tissue analyses from middle mice on day 0 (*n* = 9), day 1 (*n* = 10), day 2 (*n* = 9), day 3 (*n* = 9), day 4 (*n* = 9), and day 5 (*n* = 9) are shown. Ace, acetone; ADP, adenosine diphosphate; ATP, adenosine triphosphate; Cho, choline; Glc, glucose; Gln, glutamine; Glg, glycogen; GSH, glutathione; NAM, niacinamide; Tau, taurine.

**
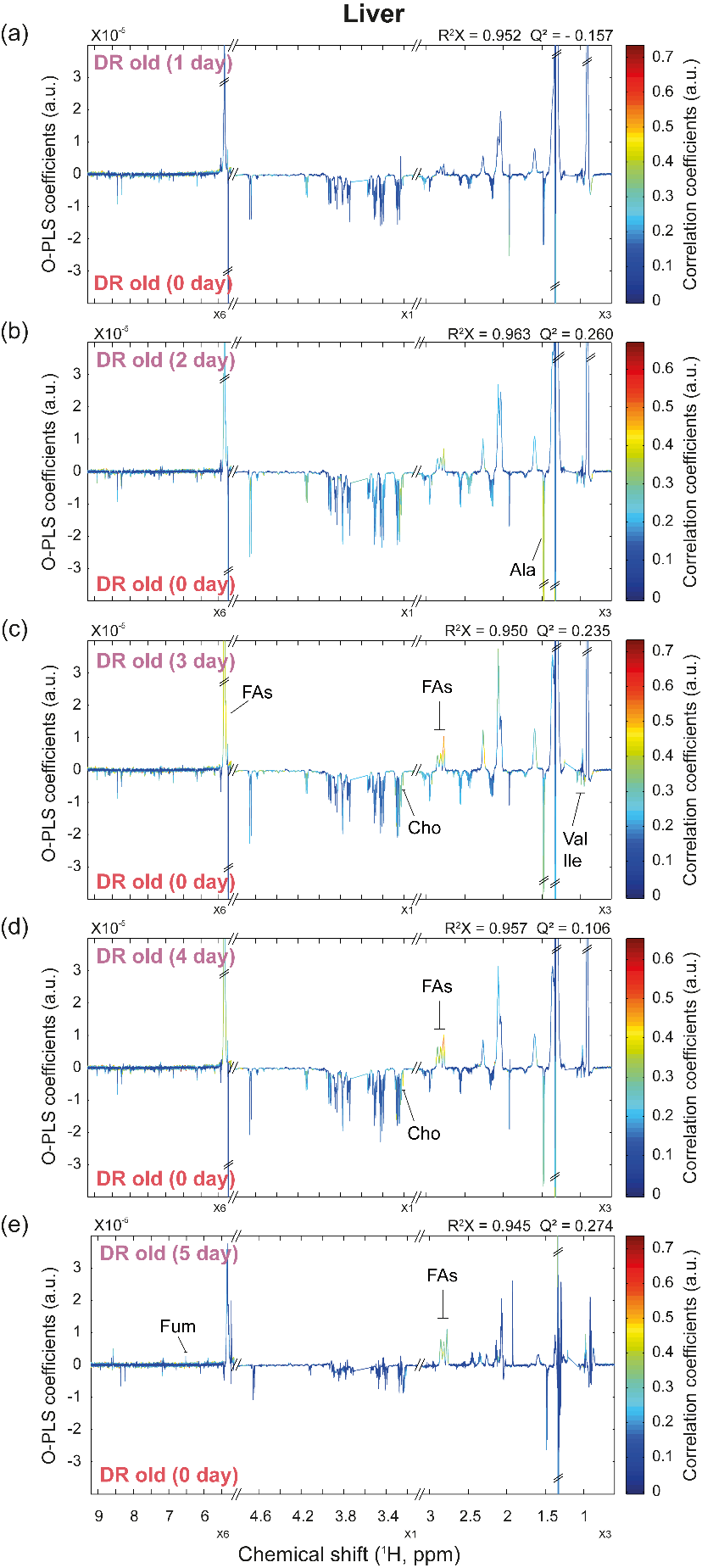
**

**FIGURE S8.** Identifications of hepatic metabolites changed in old mice during acute DR through STOCSY analysis. (a-e) STOCSY analyses that were derived from ^1^H MAS-NMR liver spectra, showing pairwise comparisons of hepatic metabolites. The liver tissue analyses from old mice on day 0 (*n* = 7), day 1 (*n* = 7), day 2 (*n* = 7), day 3 (*n* = 7), day 4 (*n* = 7), and day 5 (*n* = 7) are shown. Ala, alanine; Cho, choline; Fum, fumarate; Ile, isoleucine; Val, valine.

**
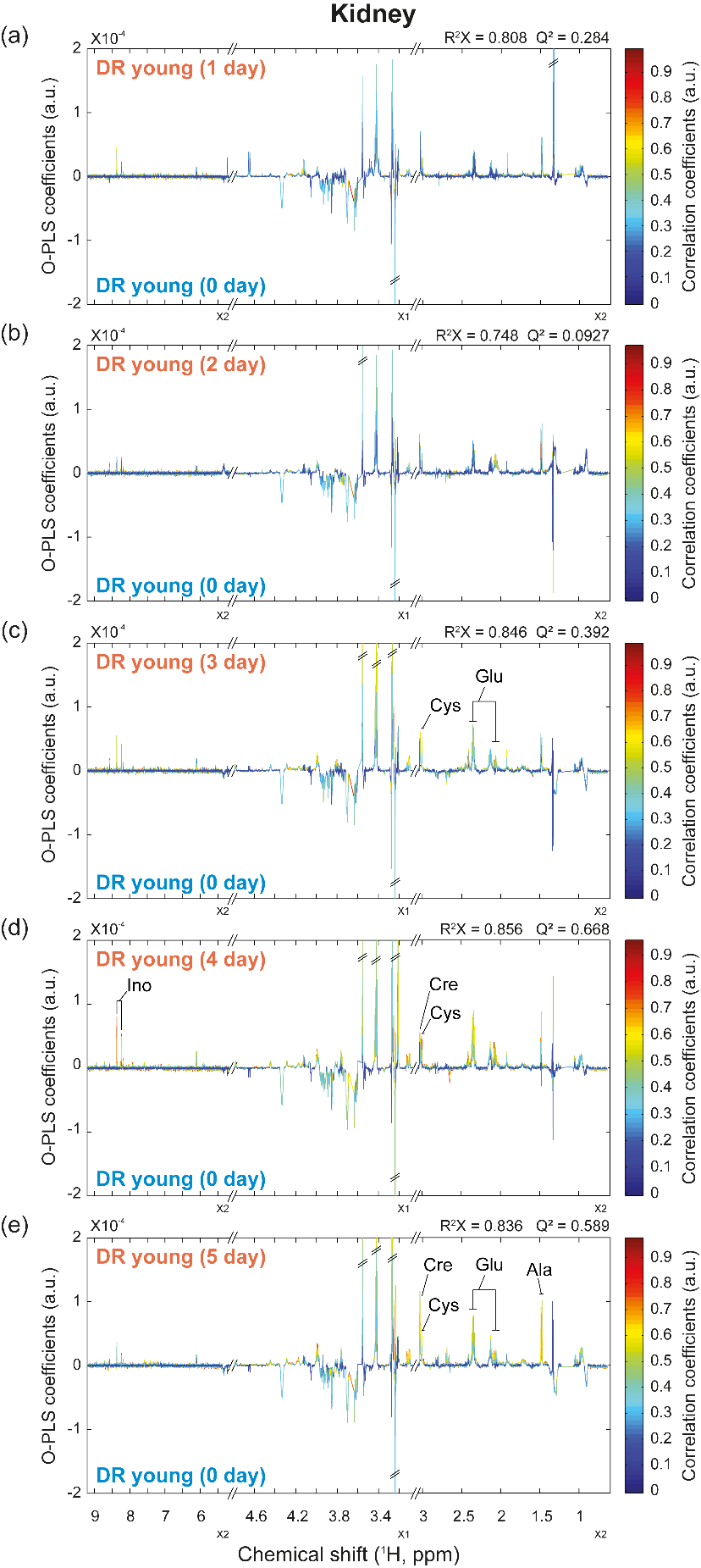
**

**FIGURE S9.** Identifications of renal metabolites changed in young mice during acute DR through STOCSY analysis. **(a-e)** STOCSY analyses that were derived from ^1^H MAS-NMR kidney spectra, showing pairwise comparisons of renal metabolites. The kidney tissue analyses from young mice on day 0 (*n* = 4), day 1 (*n* = 3), day 2 (*n* = 3), day 3 (*n* = 3), day 4 (*n* = 3), and day 5 (*n* = 3) are shown. Ala, alanine; Cre, creatine; Cys, cysteine; Glu, glutamate; Ino, inosine.


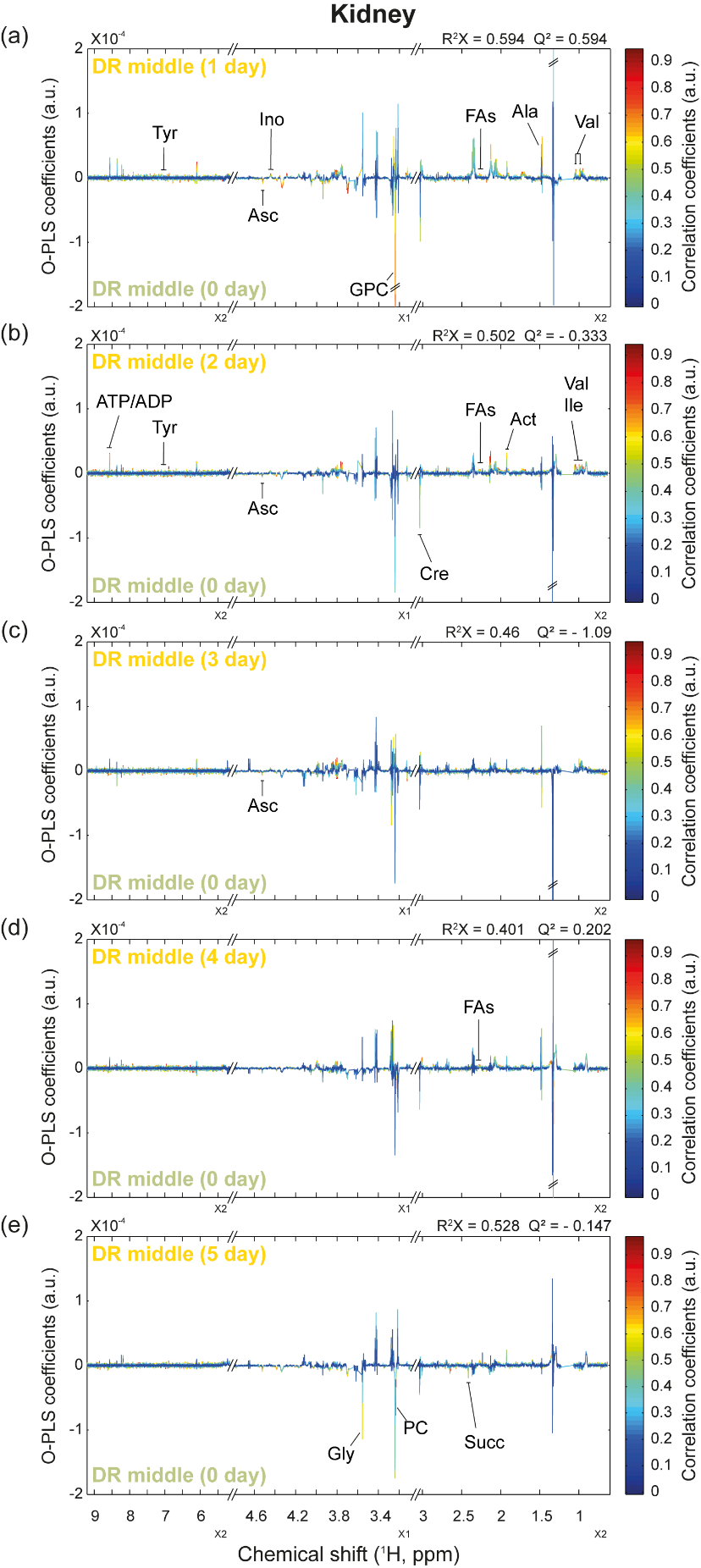


**FIGURE S10.** Identifications of renal metabolites changed in middle-aged mice during acute DR through STOCSY analysis. (a-e) STOCSY analyses that were derived from ^1^H MAS-NMR kidney spectra, showing pairwise comparisons of renal metabolites. The kidney tissue analyses from middle mice on day 0 (*n* = 4), day 1 (*n* = 5), day 2 (*n* = 4), day 3 (*n* = 4), day 4 (*n* = 4), and day 5 (*n* = 4) are shown. Act, acetate; Ala, alanine; Asc, ascorbate; ADP, adenosine diphosphate; ATP, adenosine triphosphate; Cre, creatine; GPC, glycerophosphocholine; Gly, glycine; Ile, isoleucine; Ino, inosine; PC, phosphorylcholine; Succ, succinate; Tyr, tyrosine; Val, valine


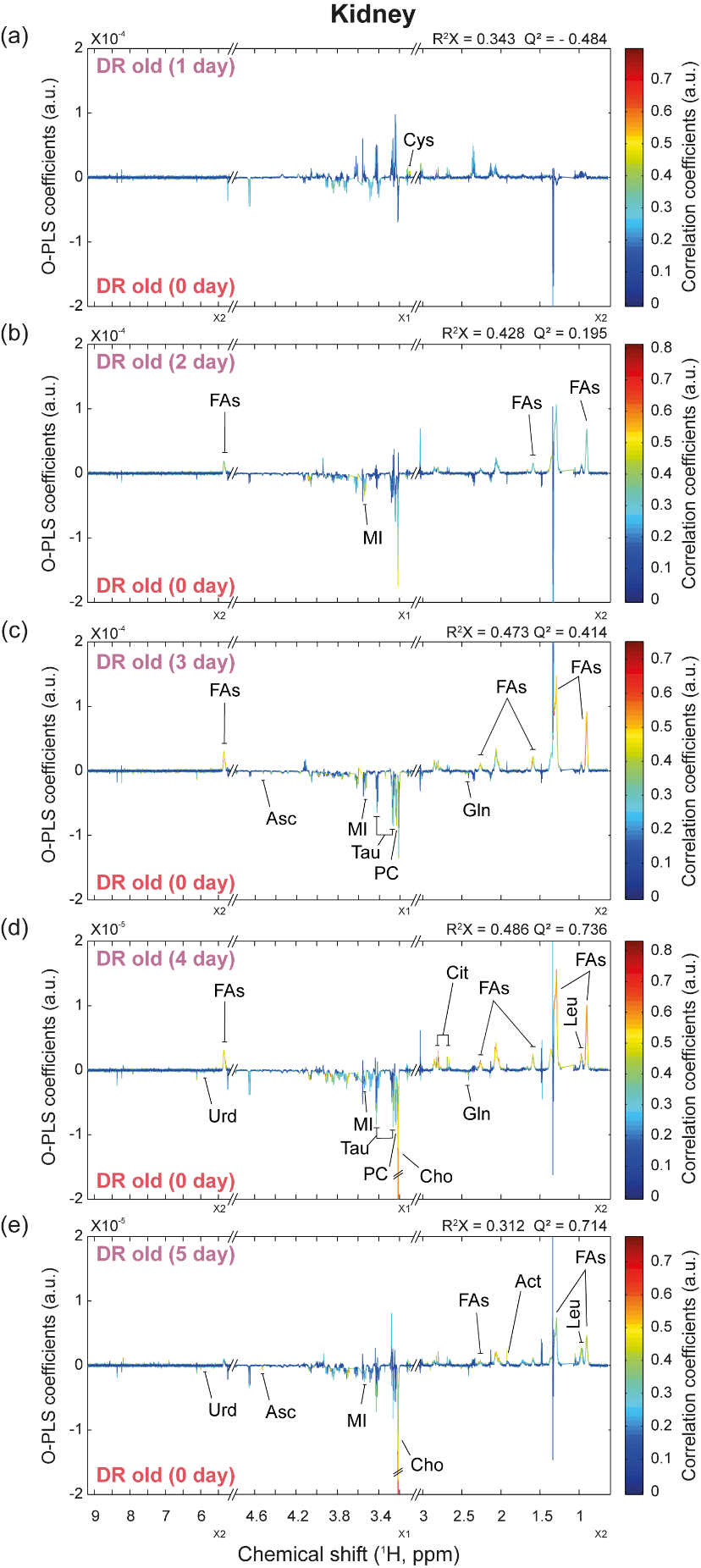


**FIGURE S11.** Identifications of renal metabolites changed in old mice during acute DR through STOCSY analysis. (a-e) STOCSY analyses that were derived from ^1^H MAS-NMR kidney spectra, showing pairwise comparisons of renal metabolites. The kidney tissue analyses from old mice on day 0 (*n* = 7), day 1 (*n* = 7), day 2 (*n* = 7), day 3 (*n* = 7), day 4 (*n* = 7), and day 5 (*n* = 7) are shown. Act, acetate; Asc, ascorbate; Cit, citrate; Cho, choline; Cys, cysteine; Gln, glutamine; Leu, leucine; MI, myo-inositol; PC, phosphorylcholine; Tau, taurine; Urd, uridine.


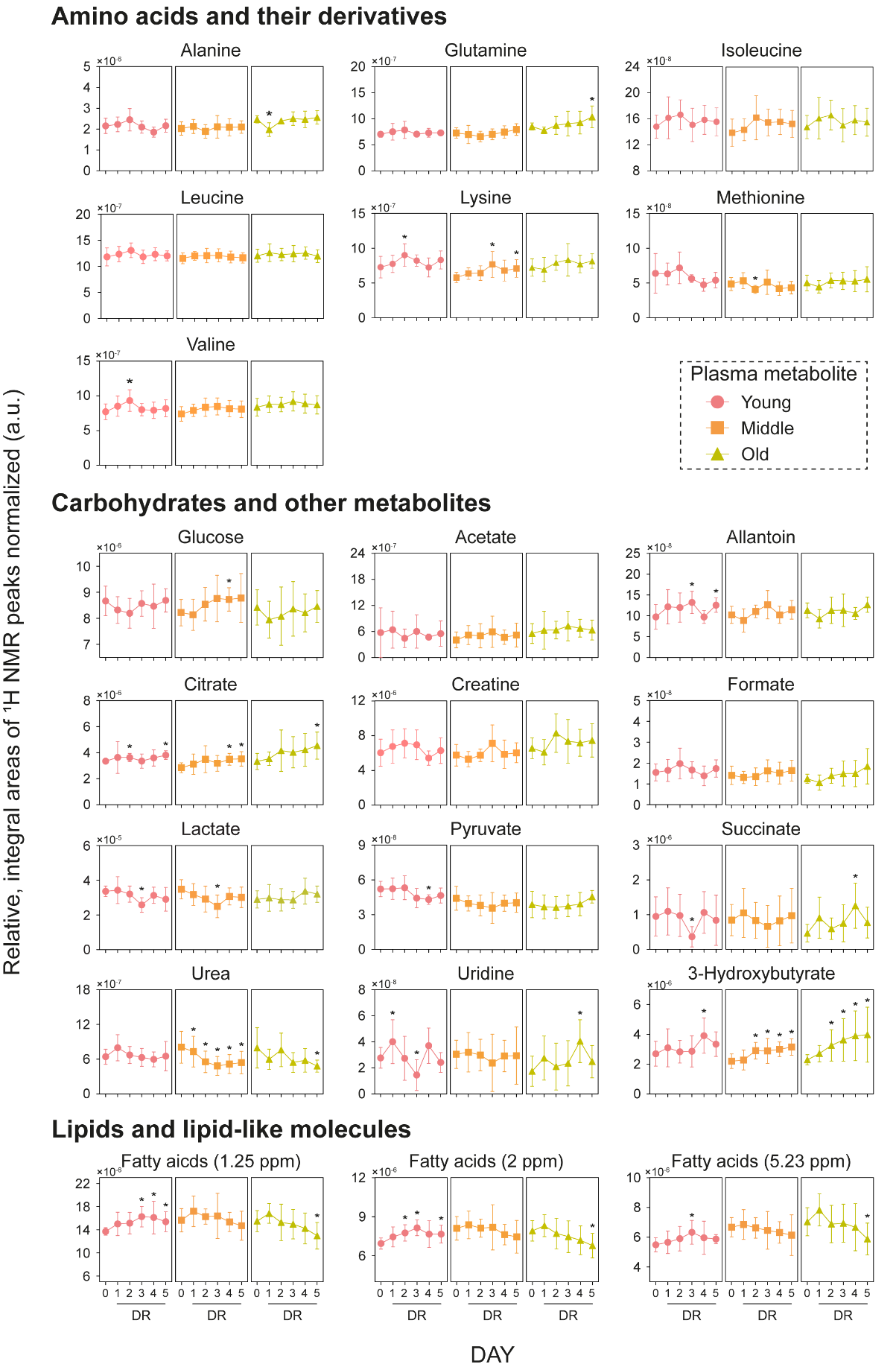


**FIGURE S12.** Relative amounts of individual plasma metabolites during acute DR for 5 days in young, middle-aged, and old mice. The symbols with pink, orange, and green colors represent the young, middle, and old mice groups, respectively. Data are presented as mean ± SD. Asterisks indicate significant differences in the levels of each plasma metabolite between AL at day 0 and DR mice of the same ages. The statistical significance of the difference in the relative amounts of plasma metabolites was determined using unpaired t-tests (**p* < 0.05).


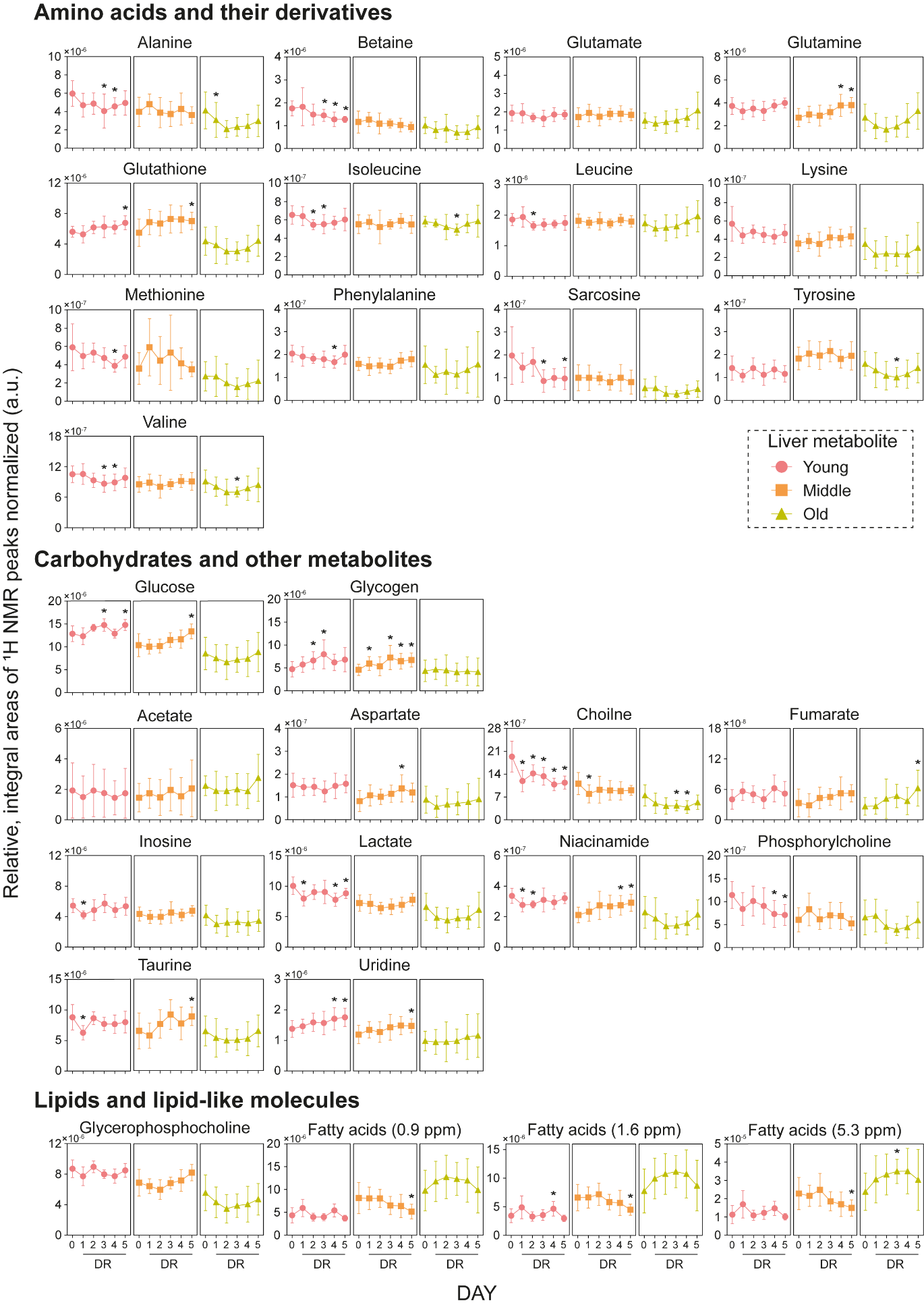


**FIGURE S13.** Relative amounts of individual liver metabolites during acute DR of 5 days in young, middle-aged, and old mice over a five-day period. The symbols with pink, orange, and green colors represent the young, middle, and old mice groups, respectively. Data are presented as mean ± SD. Asterisks indicate significant differences in the levels of each liver metabolite between AL at day 0 and DR mice of the same ages. The statistical significance of the difference in the relative amounts of liver metabolites was determined using unpaired t-tests (**p* < 0.05).


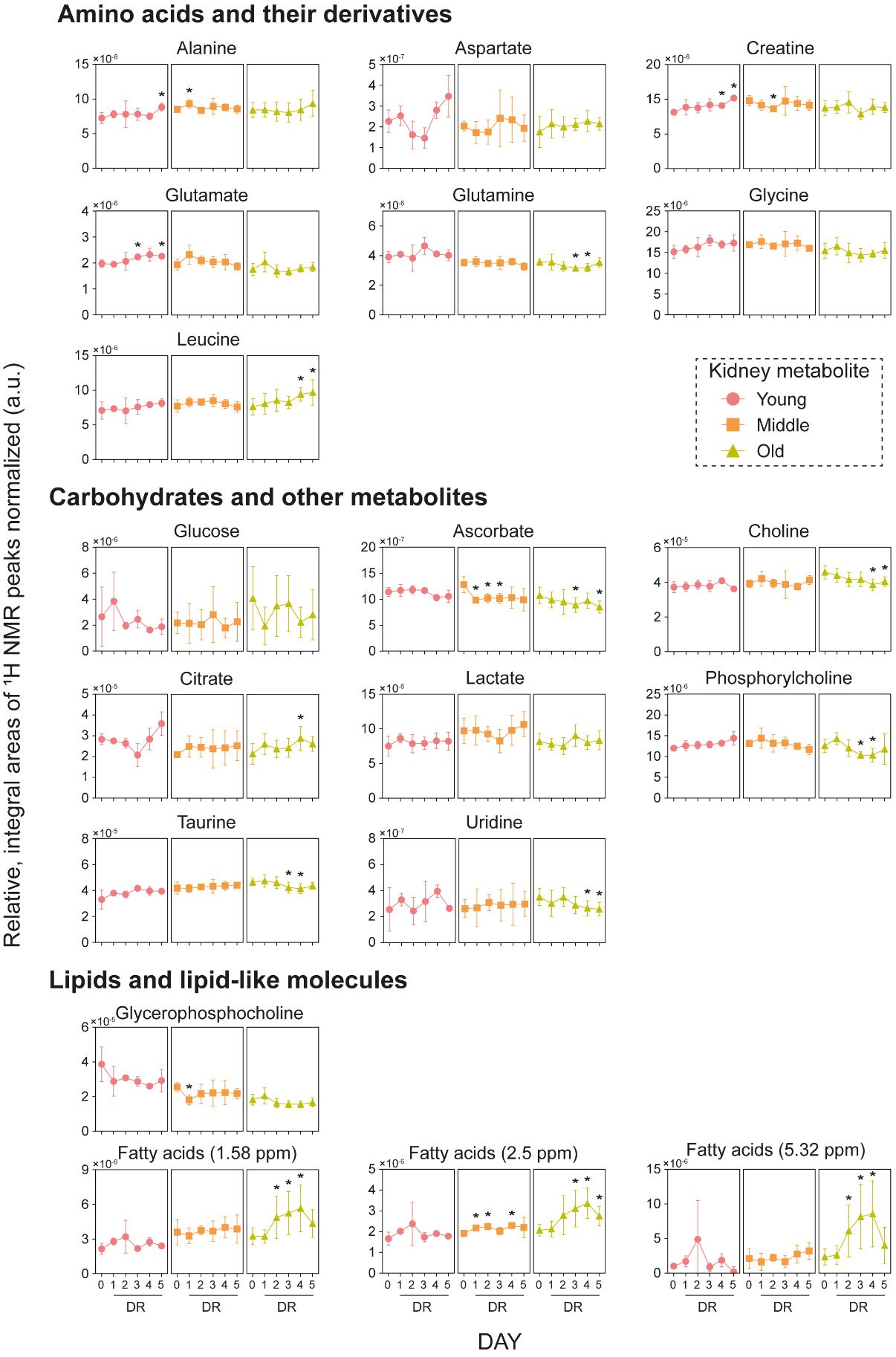


**FIGURE S14.** Relative amounts of individual kidney metabolites during acute DR of 5 days in young, middle-aged, and old mice over a five-day period. The symbols with pink, orange, and green colors represent the young, middle, and old mice groups, respectively. Data are presented as mean ± SD. Asterisks indicate significant differences in the levels of each kidney metabolite between AL at day 0 and DR mice of the same ages. The statistical significance of the difference in the relative amounts of kidney metabolites was determined using unpaired t-tests (**p* < 0.05).


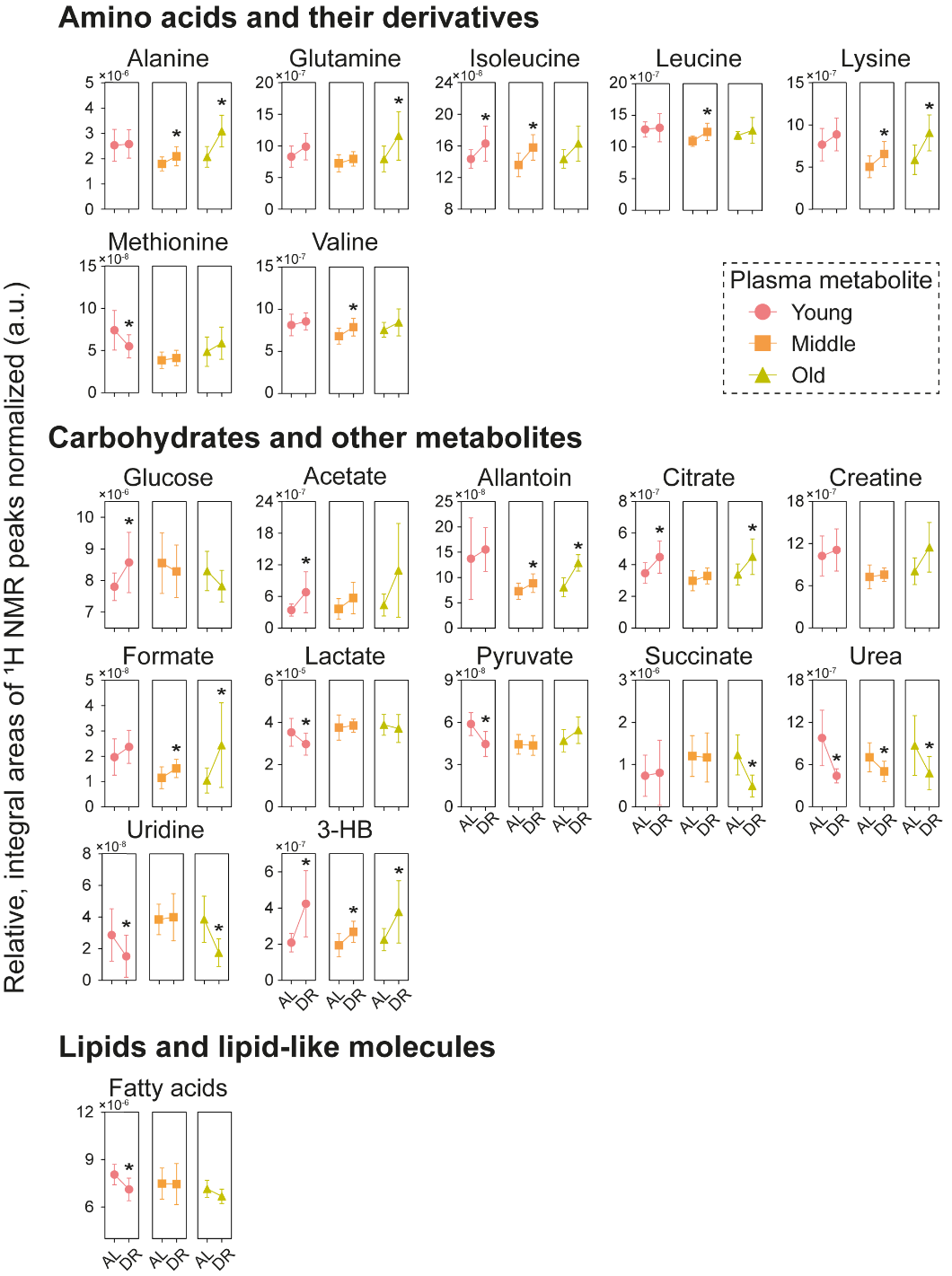


**FIGURE S15.** Relative amounts of individual plasma metabolites in young, middle-aged, and old mice fed AL and with chronic DR for 30 days. The symbols with pink, orange, and green colors represent the young, middle-aged, and old mice, respectively. Data are presented as mean ± SD. Asterisks indicate significant differences in the levels of each plasma metabolite between AL and DR mice of the same ages. The statistical significance of the difference in the relative amounts of plasma metabolites was determined using paired t-tests (*p < 0.05). 3-HB, 3-hydroxybutyrate.

**
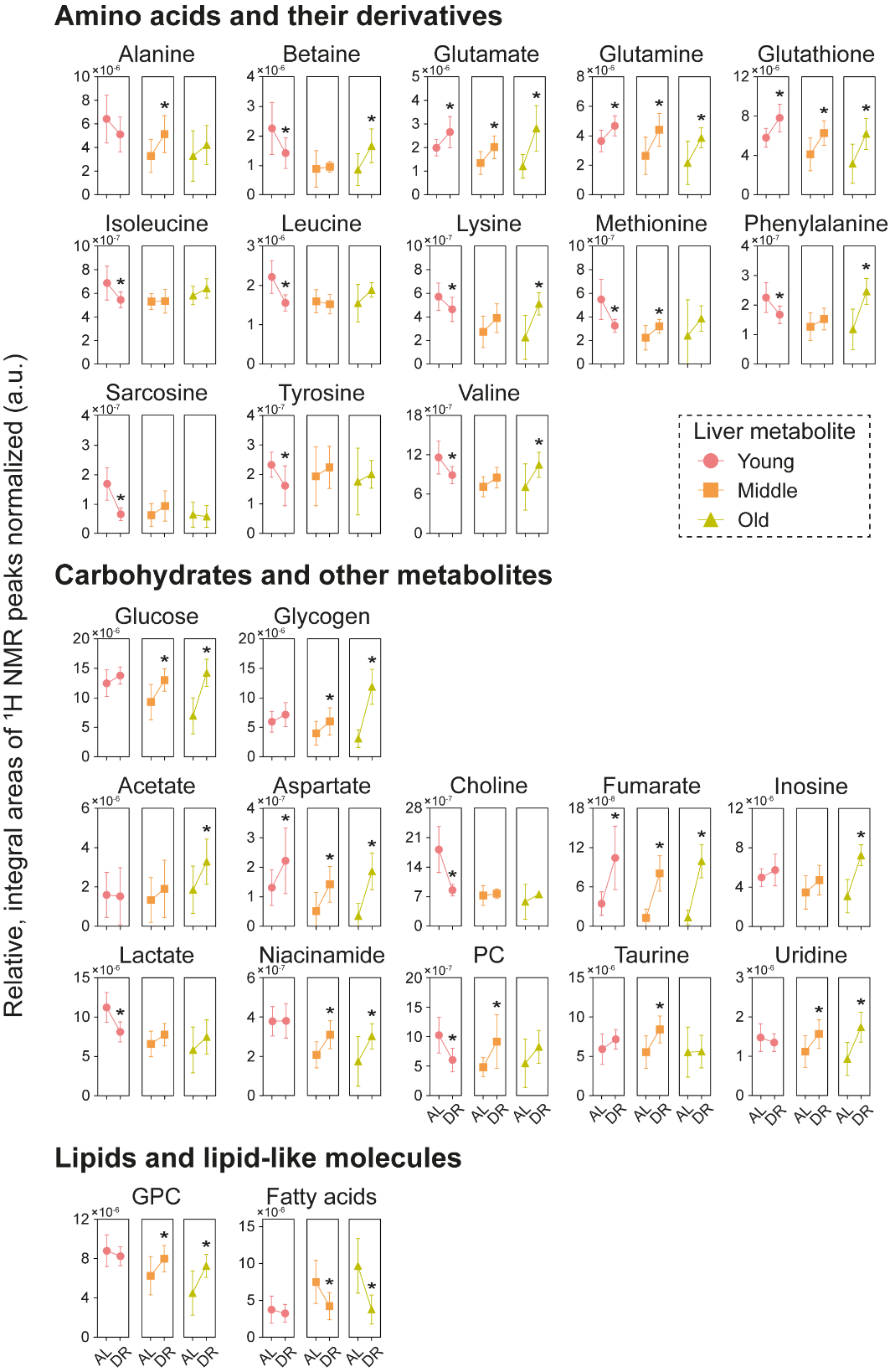
**

**FIGURE S16**. Relative amounts of individual hepatic metabolites in young, middle-aged, and old mice fed AL and with chronic DR for 30 days. The symbols with pink, orange, and green colors represent the young, middle-aged, and old mice, respectively. Data are presented as mean ± SD. Asterisks indicate significant differences in the levels of each liver metabolite between AL and DR mice of the same ages. The statistical significance of the difference in the relative amounts of liver metabolites was determined using paired t-tests (*p < 0.05). PC, phosphorylcholine; GPC, glycerophosphocholine.


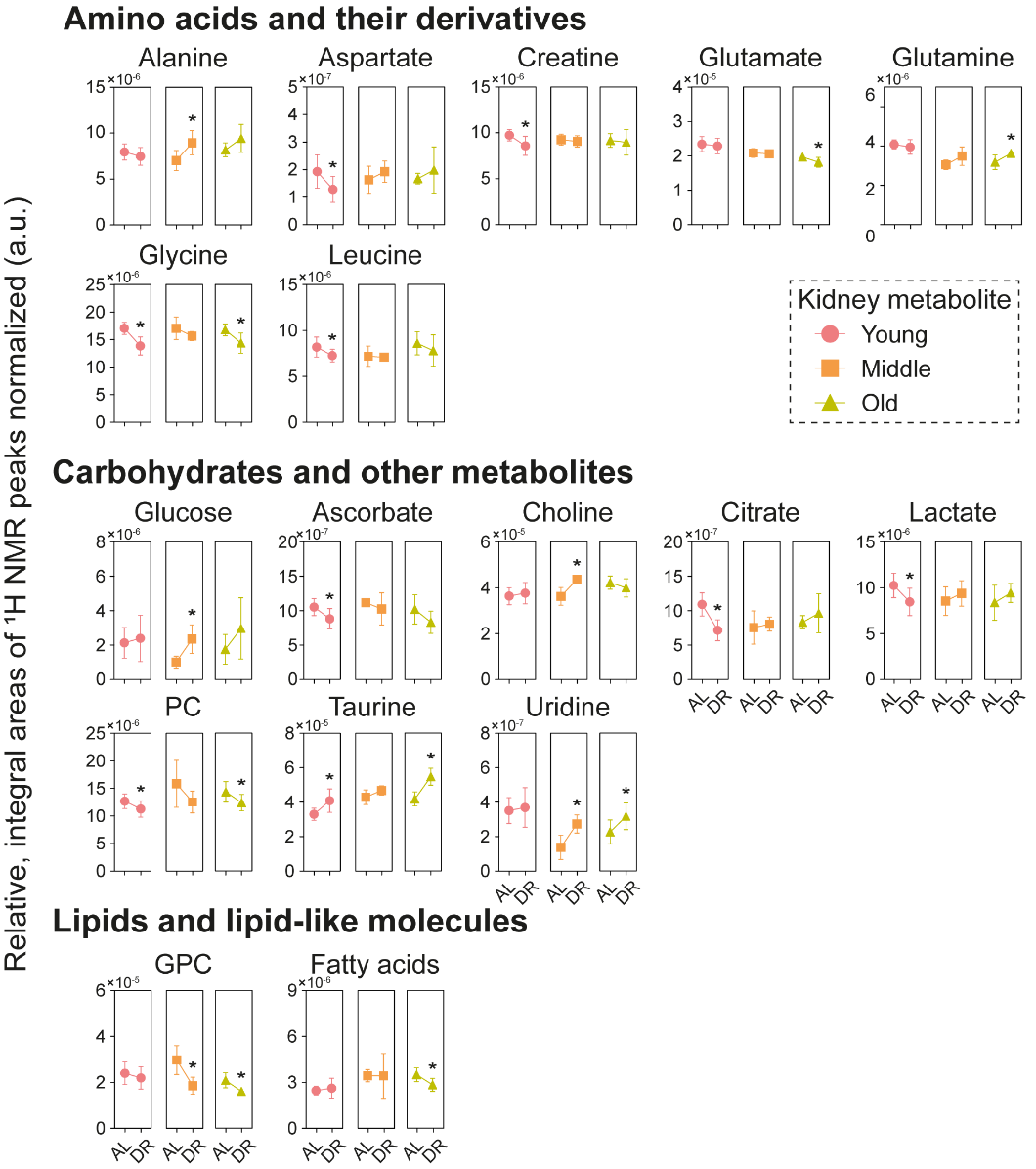


**FIGURE S17.** Relative amounts of individual renal metabolites in young, middle-aged, and old mice fed AL and with chronic DR for 30 days. The symbols with pink, orange, and green colors represent the young, middle-aged, and old mice, respectively. Data are presented as mean ± SD. Asterisks indicate significant differences in the levels of each kidney metabolite between AL and DR mice of the same ages. The statistical significance of the difference in the relative amounts of kidney metabolites was determined using paired t-tests (*p < 0.05). PC, phosphorylcholine; GPC, glycerophosphocholine.

**
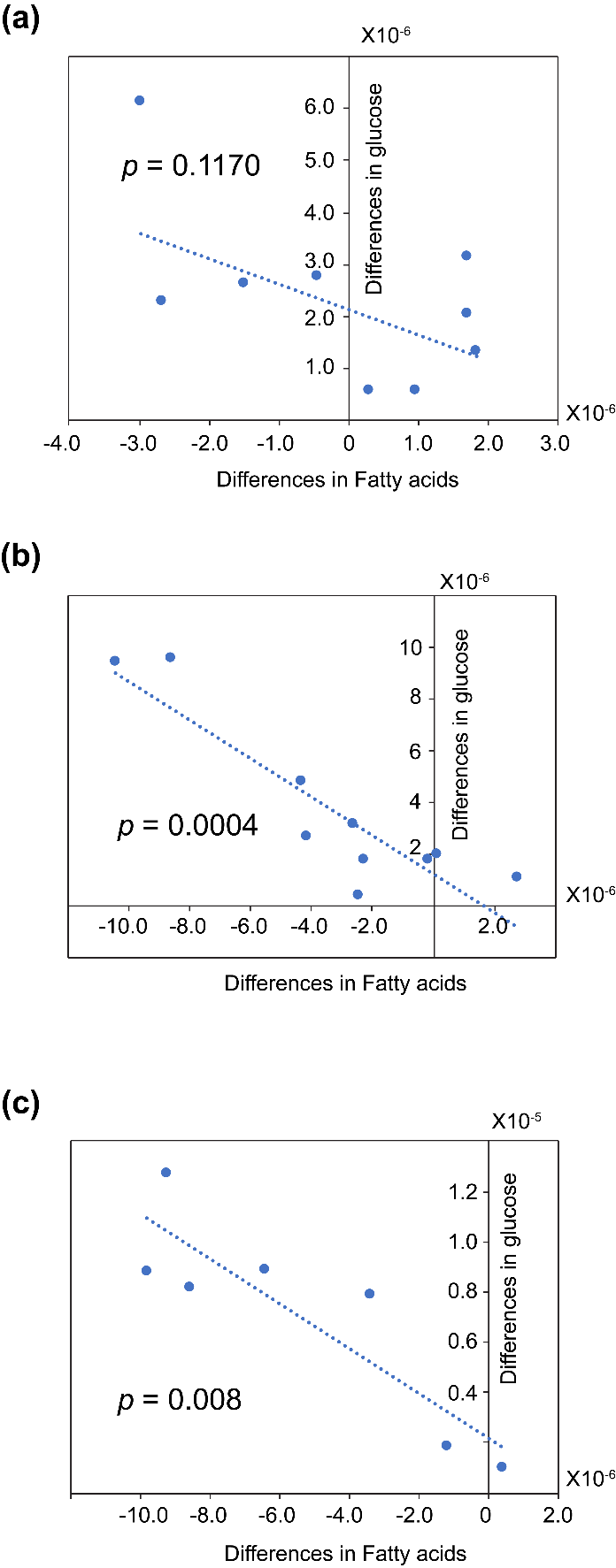
**

**FIGURE S18.** Pearson correlation between differences in fatty acids and glucose in the liver of mice during chronic DR. (a) The correlation between changes in hepatic fatty acid and glucose levels in young mice. (b) The correlation between changes in hepatic fatty acid and glucose levels in middle-aged mice. (c) The correlation between changes in hepatic fatty acid and glucose levels in old mice.

**Table S1.** Quantitative results of plasma metabolites changed after DR in young, middle-aged, and old mice.

| **Chronic DR** | **Young** | | | **Middle** | | | **Old** | | |
| --- | --- | --- | --- | --- | --- | --- | --- | --- | --- |
| **Plasma metabolite** | **AL** | **DR** | **p-value** | **AL** | **DR** | **p-value** | **AL** | **DR** | **p-value** |
| 3-Hydroxybutyrate | 2.08E-07±5.07E-08 | 4.23E-07±1.83E-07 | 2.12E-03 | 1.94E-07±6.41E-08 | 2.68E-07±5.87E-08 | 1.24E-02 | 2.25E-07±6.13E-08 | 3.79E-07±1.72E-07 | 3.41E-02 |
| Acetate | 3.39E-07±1.14E-07 | 6.78E-07±3.88E-07 | 1.63E-02 | 3.65E-07±1.93E-07 | 5.70E-07±2.98E-07 | 7.38E-02 | 4.37E-07±2.06E-07 | 1.09E-06±8.90E-07 | 6.27E-02 |
| Alanine | 2.53E-06±6.30E-07 | 2.58E-06±5.59E-07 | 8.59E-01 | 1.79E-06±2.76E-07 | 2.09E-06±3.78E-07 | 5.00E-02 | 2.07E-06±4.11E-07 | 3.08E-06±6.23E-07 | 2.25E-03 |
| Allantoin | 1.37E-07±8.04E-08 | 1.55E-07±4.34E-08 | 5.39E-01 | 7.28E-08±1.59E-08 | 8.88E-08±1.83E-08 | 4.57E-02 | 8.06E-08±1.89E-08 | 1.29E-07±1.61E-08 | 1.49E-04 |
| Citrate | 3.47E-07±6.55E-08 | 4.49E-07±1.02E-07 | 1.59E-02 | 2.99E-07±6.32E-08 | 3.30E-07±4.98E-08 | 2.39E-01 | 3.39E-07±6.64E-08 | 4.51E-07±1.13E-07 | 3.33E-02 |
| Creatine | 6.82E-07±1.89E-07 | 7.39E-07±2.00E-07 | 5.21E-01 | 4.84E-07±1.14E-07 | 5.05E-07±6.36E-08 | 6.13E-01 | 5.37E-07±1.26E-07 | 7.65E-07±2.35E-07 | 3.36E-02 |
| Formate | 1.97E-08±7.16E-09 | 2.37E-08±6.51E-09 | 2.08E-01 | 1.15E-08±4.32E-09 | 1.51E-08±3.62E-09 | 4.98E-02 | 1.04E-08±4.96E-09 | 2.44E-08±1.67E-08 | 4.15E-02 |
| Glucose | 7.80E-06±4.36E-07 | 8.57E-06±9.52E-07 | 3.26E-02 | 8.55E-06±9.57E-07 | 8.29E-06±8.32E-07 | 5.14E-01 | 8.31E-06±6.19E-07 | 7.82E-06±5.00E-07 | 1.25E-01 |
| Glutamine | 8.28E-07±1.67E-07 | 9.87E-07±2.09E-07 | 7.57E-02 | 7.24E-07±1.36E-07 | 7.94E-07±1.14E-07 | 2.22E-01 | 7.93E-07±2.03E-07 | 1.16E-06±3.83E-07 | 3.44E-02 |
| Isoleucine | 1.43E-07±1.17E-08 | 1.63E-07±2.22E-08 | 2.38E-02 | 1.36E-07±1.50E-08 | 1.58E-07±1.61E-08 | 4.12E-03 | 1.53E-07±1.31E-08 | 1.54E-07±2.90E-08 | 8.76E-01 |
| Lactate | 3.53E-05±6.58E-06 | 2.97E-05±5.24E-06 | 4.91E-02 | 3.76E-05±5.98E-06 | 3.85E-05±3.11E-06 | 6.69E-01 | 3.89E-05±4.94E-06 | 3.71E-05±6.64E-06 | 5.66E-01 |
| Leucine | 1.28E-06±1.21E-07 | 1.30E-06±2.23E-07 | 7.50E-01 | 1.09E-06±8.41E-08 | 1.24E-06±1.38E-07 | 8.62E-03 | 1.18E-06±6.27E-08 | 1.26E-06±2.03E-07 | 3.08E-01 |
| Lysine | 7.65E-07±1.91E-07 | 8.86E-07±1.95E-07 | 1.80E-01 | 5.02E-07±1.29E-07 | 6.55E-07±1.48E-07 | 2.11E-02 | 5.85E-07±1.72E-07 | 9.04E-07±2.13E-07 | 6.95E-03 |
| Methionine | 7.39E-08±2.35E-08 | 5.48E-08±1.37E-08 | 3.98E-02 | 3.85E-08±9.75E-09 | 4.12E-08±9.03E-09 | 5.31E-01 | 4.86E-08±1.74E-08 | 5.87E-08±1.91E-08 | 3.04E-01 |
| Phenylalanine | 8.77E-08±1.87E-08 | 7.28E-08±1.10E-08 | 4.40E-02 | 6.72E-08±1.20E-08 | 6.66E-08±8.86E-09 | 8.87E-01 | 7.38E-08±7.60E-09 | 7.40E-08±2.85E-08 | 9.87E-01 |
| Pyruvate | 5.89E-07±8.13E-08 | 4.46E-07±8.89E-08 | 1.49E-03 | 4.44E-07±6.96E-08 | 4.37E-07±6.99E-08 | 8.01E-01 | 4.70E-07±7.91E-08 | 5.45E-07±9.46E-08 | 1.17E-01 |
| Succinate | 7.33E-07±4.88E-07 | 8.02E-07±7.72E-07 | 8.13E-01 | 1.20E-06±4.84E-07 | 1.17E-06±5.83E-07 | 8.86E-01 | 1.23E-06±4.75E-07 | 4.92E-07±2.60E-07 | 2.96E-03 |
| Urea | 9.76E-07±3.95E-07 | 4.36E-07±1.00E-07 | 5.57E-04 | 7.03E-07±2.06E-07 | 5.03E-07±1.43E-07 | 1.93E-02 | 8.70E-07±4.26E-07 | 4.75E-07±2.38E-07 | 4.92E-02 |
| Uridine | 2.86E-08±1.66E-08 | 1.30E-08±1.64E-08 | 4.95E-02 | 3.85E-08±9.68E-09 | 3.99E-08±1.49E-08 | 7.94E-01 | 3.86E-08±1.46E-08 | 1.75E-08±8.77E-09 | 5.50E-03 |
| Valine | 8.13E-07±1.27E-07 | 8.55E-07±1.02E-07 | 4.22E-01 | 6.81E-07±9.53E-08 | 7.88E-07±1.05E-07 | 2.41E-02 | 7.56E-07±8.77E-08 | 8.44E-07±1.60E-07 | 1.98E-01 |
| Fattyacids (1.25) | 1.54E-05±1.81E-06 | 1.34E-05±2.34E-06 | 4.23E-02 | 1.48E-05±2.02E-06 | 1.49E-05±3.07E-06 | 9.88E-01 | 1.43E-05±1.04E-06 | 1.37E-05±1.58E-06 | 3.89E-01 |
| Fattyacids (2) | 8.04E-06±6.36E-07 | 7.10E-06±7.19E-07 | 6.28E-03 | 7.48E-06±9.86E-07 | 7.45E-06±1.30E-06 | 9.56E-01 | 7.15E-06±5.38E-07 | 6.68E-06±4.57E-07 | 9.41E-02 |
| Fattyacids (5.23) | 6.51E-06±1.03E-06 | 5.00E-06±9.79E-07 | 3.59E-03 | 6.30E-06±7.31E-07 | 5.79E-06±1.08E-06 | 2.18E-01 | 6.57E-06±7.29E-07 | 5.67E-06±1.26E-06 | 1.05E-01 |

The data is presented as Mean ± SD.

**Table S2.** Quantitative results of hepatic metabolites changed after DR in young, middle-aged, and old mice.

| **Chronic DR** | **Young** | | | **Middle** | | | **Old** | | |
| --- | --- | --- | --- | --- | --- | --- | --- | --- | --- |
| **Hepatic metabolite** | **AL** | **DR** | **p-value** | **AL** | **DR** | **p-value** | **AL** | **DR** | **p-value** |
| Acetate | 1.59E-06±1.14E-06 | 1.52E-06±1.45E-06 | 9.04E-01 | 1.34E-06±1.13E-06 | 1.90E-06±1.45E-06 | 3.29E-01 | 1.87E-06±1.20E-06 | 3.30E-06±1.15E-06 | 3.57E-02 |
| Alanine | 6.42E-06±2.04E-06 | 5.11E-06±1.47E-06 | 1.16E-01 | 3.29E-06±1.40E-06 | 5.14E-06±1.57E-06 | 1.01E-02 | 3.28E-06±2.14E-06 | 4.21E-06±1.65E-06 | 3.67E-01 |
| Ascorbate | 6.69E-07±9.12E-08 | 1.47E-02±4.88E-02 | 1.62E-01 | 4.00E-07±1.34E-07 | 8.65E-04±2.87E-03 | 9.51E-03 | 3.38E-07±2.07E-07 | 3.72E-02±1.05E-01 | 2.98E-01 |
| Aspartate | 1.31E-07±6.01E-08 | 2.22E-07±1.11E-07 | 3.50E-02 | 5.03E-08±6.44E-08 | 1.42E-07±6.05E-08 | 3.28E-03 | 3.29E-08±4.46E-08 | 1.86E-07±6.18E-08 | 9.06E-05 |
| ATP/ADP | 6.00E-07±1.22E-07 | 5.79E-02±1.92E-01 | 6.37E-01 | 3.80E-07±9.55E-08 | 2.09E-03±6.94E-03 | 2.30E-02 | 3.73E-07±2.59E-07 | 2.09E-02±5.90E-02 | 1.67E-01 |
| Betaine | 2.26E-06±8.79E-07 | 1.42E-06±5.19E-07 | 1.83E-02 | 8.83E-07±6.21E-07 | 9.56E-07±1.75E-07 | 7.25E-01 | 8.42E-07±5.41E-07 | 1.65E-06±5.75E-07 | 1.51E-02 |
| Choline | 1.82E-06±5.45E-07 | 8.55E-07±1.36E-07 | 3.71E-05 | 7.26E-07±2.36E-07 | 7.71E-07±1.11E-07 | 5.93E-01 | 5.79E-07±4.14E-07 | 7.54E-07±6.41E-08 | 2.92E-01 |
| Fumarate | 3.46E-08±1.79E-08 | 1.04E-07±4.83E-08 | 4.53E-04 | 1.16E-08±1.48E-08 | 8.07E-08±2.71E-08 | 5.79E-07 | 1.34E-08±1.12E-08 | 9.94E-08±2.55E-08 | 9.13E-07 |
| Glucose | 1.25E-05±2.26E-06 | 1.38E-05±1.47E-06 | 1.40E-01 | 9.29E-06±2.99E-06 | 1.30E-05±1.91E-06 | 3.26E-03 | 6.95E-06±3.05E-06 | 1.42E-05±2.31E-06 | 1.85E-04 |
| Glutamate | 1.99E-06±3.46E-07 | 2.65E-06±6.65E-07 | 1.18E-02 | 1.34E-06±4.83E-07 | 2.02E-06±4.79E-07 | 4.70E-03 | 1.20E-06±5.06E-07 | 2.81E-06±9.62E-07 | 1.17E-03 |
| Glutamine | 3.64E-06±7.29E-07 | 4.67E-06±6.81E-07 | 4.36E-03 | 2.63E-06±1.26E-06 | 4.40E-06±1.11E-06 | 3.13E-03 | 2.16E-06±1.46E-06 | 3.86E-06±6.83E-07 | 1.46E-02 |
| Glutathione | 5.79E-06±9.54E-07 | 7.81E-06±1.40E-06 | 1.42E-03 | 4.11E-06±1.67E-06 | 6.28E-06±1.25E-06 | 3.44E-03 | 3.16E-06±2.00E-06 | 6.20E-06±1.57E-06 | 6.45E-03 |
| Glycerophosphocholine | 8.80E-06±1.61E-06 | 8.24E-06±9.64E-07 | 3.61E-01 | 6.26E-06±1.94E-06 | 8.00E-06±1.34E-06 | 2.87E-02 | 4.49E-06±2.25E-06 | 7.25E-06±1.17E-06 | 1.22E-02 |
| Glycogen | 5.96E-06±1.77E-06 | 7.15E-06±2.03E-06 | 1.79E-01 | 4.00E-06±2.01E-06 | 6.02E-06±2.30E-06 | 4.47E-02 | 3.10E-06±1.50E-06 | 1.19E-05±2.95E-06 | 5.10E-06 |
| Inosine | 5.00E-07±9.01E-08 | 5.74E-07±1.61E-07 | 2.22E-01 | 3.47E-07±1.69E-07 | 4.72E-07±1.51E-07 | 9.18E-02 | 3.09E-07±1.70E-07 | 7.27E-07±1.07E-07 | 8.62E-05 |
| Isoleucine | 6.87E-07±1.44E-07 | 5.45E-07±6.75E-08 | 1.11E-02 | 5.29E-07±6.83E-08 | 5.33E-07±1.01E-07 | 9.13E-01 | 5.81E-07±7.79E-08 | 6.40E-07±8.27E-08 | 1.77E-01 |
| Lactate | 1.12E-05±1.90E-06 | 8.11E-06±1.27E-06 | 4.26E-04 | 6.59E-06±1.61E-06 | 7.76E-06±1.43E-06 | 9.37E-02 | 5.82E-06±2.91E-06 | 7.48E-06±2.17E-06 | 2.39E-01 |
| Leucine | 2.22E-06±4.16E-07 | 1.56E-06±2.05E-07 | 2.77E-04 | 1.60E-06±2.94E-07 | 1.52E-06±2.47E-07 | 5.51E-01 | 1.55E-06±4.78E-07 | 1.88E-06±1.82E-07 | 1.05E-01 |
| Lysine | 5.71E-07±1.15E-07 | 4.64E-07±1.03E-07 | 4.12E-02 | 2.73E-07±1.33E-07 | 3.90E-07±1.21E-07 | 5.03E-02 | 2.26E-07±1.86E-07 | 5.11E-07±9.49E-08 | 2.89E-03 |
| Methionine | 5.48E-07±1.71E-07 | 3.25E-07±5.51E-08 | 9.71E-04 | 2.23E-07±1.04E-07 | 3.20E-07±5.85E-08 | 1.79E-02 | 2.41E-07±3.04E-07 | 3.86E-07±1.09E-07 | 2.54E-01 |
| Niacinamide | 3.78E-07±7.49E-08 | 3.80E-07±8.74E-08 | 9.48E-01 | 2.08E-07±6.63E-08 | 3.10E-07±7.13E-08 | 2.97E-03 | 1.74E-07±1.26E-07 | 3.02E-07±6.42E-08 | 3.09E-02 |
| Phenylalanine | 2.25E-07±5.07E-08 | 1.67E-07±2.98E-08 | 5.90E-03 | 1.27E-07±4.68E-08 | 1.53E-07±3.66E-08 | 1.76E-01 | 1.18E-07±6.88E-08 | 2.46E-07±4.40E-08 | 9.86E-04 |
| Phosphorylcholine | 1.02E-06±3.03E-07 | 6.04E-07±2.00E-07 | 1.82E-03 | 4.81E-07±1.60E-07 | 9.15E-07±4.53E-07 | 7.68E-03 | 5.48E-07±4.10E-07 | 8.25E-07±2.79E-07 | 1.56E-01 |
| Sarcosine | 1.69E-07±5.53E-08 | 6.59E-08±2.14E-08 | 3.31E-05 | 6.27E-08±3.88E-08 | 9.37E-08±5.13E-08 | 1.32E-01 | 6.42E-08±4.23E-08 | 5.82E-08±3.69E-08 | 7.76E-01 |
| Taurine | 5.91E-06±1.95E-06 | 7.16E-06±1.25E-06 | 1.05E-01 | 5.53E-06±2.07E-06 | 8.41E-06±1.71E-06 | 2.59E-03 | 5.53E-06±3.16E-06 | 5.59E-06±2.07E-06 | 9.64E-01 |
| Tyrosine | 2.33E-07±4.25E-08 | 1.61E-07±6.75E-08 | 1.14E-02 | 1.94E-07±1.00E-07 | 2.24E-07±7.13E-08 | 4.43E-01 | 1.76E-07±1.13E-07 | 2.00E-07±4.71E-08 | 6.04E-01 |
| Uridine | 1.48E-07±3.51E-08 | 1.35E-07±2.27E-08 | 3.54E-01 | 1.12E-07±4.02E-08 | 1.57E-07±3.58E-08 | 1.45E-02 | 9.32E-08±4.18E-08 | 1.74E-07±3.80E-08 | 1.90E-03 |
| Valine | 1.16E-06±2.54E-07 | 8.89E-07±1.32E-07 | 7.95E-03 | 7.12E-07±1.55E-07 | 8.51E-07±1.54E-07 | 5.47E-02 | 8.13E-07±2.12E-07 | 1.05E-06±1.92E-07 | 4.58E-02 |
| Fattyacids (0.9) | 4.45E-05±2.09E-05 | 4.22E-05±1.66E-05 | 7.90E-01 | 9.92E-05±3.86E-05 | 5.37E-05±2.56E-05 | 5.33E-03 | 1.23E-04±4.92E-05 | 4.35E-05±2.56E-05 | 1.99E-03 |
| Fattyacids (1.6) | 3.74E-06±1.85E-06 | 3.23E-06±1.21E-06 | 4.75E-01 | 7.50E-06±2.92E-06 | 4.21E-06±1.84E-06 | 6.57E-03 | 9.68E-06±3.70E-06 | 3.77E-06±1.98E-06 | 2.34E-03 |
| Fattyacids (5.3) | 1.27E-05±6.68E-06 | 1.06E-05±4.33E-06 | 4.11E-01 | 2.61E-05±9.39E-06 | 1.48E-05±6.96E-06 | 5.76E-03 | 3.09E-05±1.03E-05 | 1.28E-05±7.59E-06 | 2.09E-03 |

The data is presented as Mean ± SD.

**Table S3.** Quantitative results of renal metabolites changed after DR in young, middle-aged, and old mice.

| **Chronic DR** | **Young** | | | **Middle** | | | **Old** | | |
| --- | --- | --- | --- | --- | --- | --- | --- | --- | --- |
| **Renal metabolite** | **AL** | **DR** | **p-value** | **AL** | **DR** | **p-value** | **AL** | **DR** | **p-value** |
| Alanine | 7.95E-06±8.78E-07 | 7.46E-06±9.72E-07 | 2.85E-01 | 7.02E-06±1.08E-06 | 8.95E-06±1.30E-06 | 2.48E-02 | 8.18E-06±7.57E-07 | 9.43E-06±1.52E-06 | 6.05E-02 |
| Ascorbate | 1.05E-06±1.23E-07 | 8.83E-07±1.50E-07 | 1.80E-02 | 1.12E-06±5.71E-08 | 1.03E-06±2.32E-07 | 3.65E-01 | 1.02E-06±2.12E-07 | 8.33E-07±1.61E-07 | 8.27E-02 |
| Aspartate | 2.22E-07±7.17E-08 | 1.50E-07±5.53E-08 | 3.00E-02 | 1.84E-07±5.15E-08 | 2.26E-07±3.89E-08 | 1.73E-01 | 2.01E-07±1.85E-08 | 2.29E-07±9.23E-08 | 4.07E-01 |
| Choline | 3.64E-05±3.72E-06 | 3.76E-05±4.66E-06 | 5.34E-01 | 3.63E-05±3.86E-06 | 4.36E-05±1.88E-06 | 3.80E-03 | 4.22E-05±2.89E-06 | 3.99E-05±4.00E-06 | 2.22E-01 |
| Citrate | 1.09E-06±1.67E-07 | 7.16E-07±1.54E-07 | 1.47E-04 | 7.55E-07±2.39E-07 | 8.04E-07±1.02E-07 | 6.78E-01 | 8.32E-07±9.55E-08 | 9.63E-07±2.85E-07 | 2.42E-01 |
| Creatine | 9.75E-06±6.11E-07 | 8.59E-06±1.03E-06 | 1.05E-02 | 9.24E-06±5.89E-07 | 9.07E-06±6.14E-07 | 6.50E-01 | 9.18E-06±7.61E-07 | 8.98E-06±1.39E-06 | 7.34E-01 |
| Glucose | 2.13E-06±8.89E-07 | 2.39E-06±1.35E-06 | 6.41E-01 | 1.01E-06±3.44E-07 | 2.35E-06±8.30E-07 | 5.52E-03 | 1.76E-06±8.61E-07 | 2.97E-06±1.77E-06 | 1.09E-01 |
| Glutamate | 2.34E-05±2.16E-06 | 2.29E-05±2.25E-06 | 6.07E-01 | 2.08E-05±1.26E-06 | 2.06E-05±7.84E-07 | 7.33E-01 | 1.96E-05±9.37E-07 | 1.82E-05±1.40E-06 | 2.96E-02 |
| Glutamine | 4.08E-06±2.31E-07 | 3.95E-06±3.71E-07 | 3.89E-01 | 3.05E-06±2.44E-07 | 3.48E-06±4.74E-07 | 7.85E-02 | 3.18E-06±3.66E-07 | 3.62E-06±1.92E-07 | 1.23E-02 |
| Glycerophosphocholine | 2.40E-05±4.95E-06 | 2.19E-05±4.89E-06 | 3.95E-01 | 2.97E-05±6.32E-06 | 1.85E-05±3.73E-06 | 6.97E-03 | 2.08E-05±3.27E-06 | 1.60E-05±1.63E-06 | 3.71E-03 |
| Glycine | 1.71E-05±1.08E-06 | 1.38E-05±1.65E-06 | 1.59E-04 | 1.70E-05±2.05E-06 | 1.57E-05±7.97E-07 | 1.90E-01 | 1.68E-05±1.08E-06 | 1.44E-05±1.83E-06 | 7.85E-03 |
| Lactate | 1.03E-05±1.34E-06 | 8.46E-06±1.49E-06 | 1.60E-02 | 8.54E-06±1.54E-06 | 9.37E-06±1.38E-06 | 3.73E-01 | 8.38E-06±1.91E-06 | 9.43E-06±1.03E-06 | 2.15E-01 |
| Leucine | 8.19E-06±1.08E-06 | 7.27E-06±6.70E-07 | 4.34E-02 | 7.19E-06±1.11E-06 | 7.10E-06±2.78E-07 | 8.56E-01 | 8.59E-06±1.26E-06 | 7.79E-06±1.69E-06 | 3.12E-01 |
| Phosphorylcholine | 1.27E-05±1.33E-06 | 1.12E-05±1.48E-06 | 4.58E-02 | 1.58E-05±4.26E-06 | 1.25E-05±1.98E-06 | 1.50E-01 | 1.43E-05±1.87E-06 | 1.24E-05±1.51E-06 | 4.71E-02 |
| Taurine | 3.30E-05±3.52E-06 | 4.09E-05±6.80E-06 | 6.63E-03 | 4.29E-05±4.23E-06 | 4.68E-05±2.75E-06 | 1.11E-01 | 4.18E-05±4.15E-06 | 5.48E-05±4.91E-06 | 9.47E-05 |
| Uridine | 3.51E-07±7.48E-08 | 3.69E-07±1.15E-07 | 7.05E-01 | 1.38E-07±7.14E-08 | 2.74E-07±5.26E-08 | 6.44E-03 | 2.26E-07±6.90E-08 | 3.18E-07±7.79E-08 | 3.13E-02 |
| Fattyacids (0.8) | 6.90E-06±1.58E-06 | 7.54E-06±9.87E-07 | 3.17E-01 | 1.00E-05±2.68E-06 | 6.07E-06±9.96E-07 | 1.28E-02 | 1.09E-05±1.80E-06 | 7.31E-06±1.80E-06 | 1.83E-03 |
| Fattyacids (1.58) | 2.47E-06±2.94E-07 | 2.62E-06±6.50E-07 | 5.36E-01 | 3.44E-06±3.98E-07 | 3.42E-06±1.46E-06 | 9.84E-01 | 3.49E-06±4.43E-07 | 2.83E-06±4.23E-07 | 1.13E-02 |
| Fattyacids (2.5) | 2.09E-06±2.44E-07 | 2.16E-06±4.90E-07 | 7.11E-01 | 2.12E-06±2.71E-07 | 1.83E-06±2.77E-07 | 1.16E-01 | 2.42E-06±2.92E-07 | 2.08E-06±2.07E-07 | 2.41E-02 |
| Fattyacids (5.32) | 1.64E-06±6.24E-07 | 1.78E-06±1.61E-06 | 8.05E-01 | 3.79E-06±1.52E-06 | 9.00E-07±3.85E-07 | 2.68E-03 | 3.49E-06±1.29E-06 | 2.73E-06±1.86E-06 | 3.69E-01 |

The data is presented as Mean ± SD.
