# Supplementary material for: Age‐Dependent Metabolomic Signatures of Dietary Restriction in Mice
Source: Aging Cell. 2025 Dec 1;25(1):e70309. doi: 10.1111/acel.70309 (PMC12740100; doi:10.1111/acel.70309)
Supplement: Supplementary file 2 — Table S1: Quantitative results of plasma metabolites changed after DR in young, middle‐aged, and old mice. Table S2: Quantitative results of hepatic metabolites changed after DR in young, middle‐aged, and old mice. Table S3: Quantitative results of renal metabolites changed after DR in young, middle‐aged, and old mice. [file ACEL-25-e70309-s002.docx]

**AGING CELL AUTHOR CHECKLIST**. ***Authors should submit this checklist together with their manuscript. Please ensure that you have read the Author Guidelines in detail before submission.***

| **Title** | **Age-Dependent Metabolomic Signatures of Dietary Restriction in Mice** | | | | | | |
| --- | --- | --- | --- | --- | --- | --- | --- |
| **Authors** | Ji-sue Lee, Vindya H. J. Hetti Arachchige, Eun-Hee Kim, Eunjung Bang, Young-Shick Hong | | | | | | |
| **Manuscript Type** | Research article | | | | | | |
| **Total Character Count (including spaces)^1^** | 43,235 | | | | | | |
| **Word count of Summary^2^** | 241 | | | | | | |
| **Number of papers cited in the References^3^** | 55 | | | | | | |
| **Listing of all Tables (Table1, Table 2 etc)^4^** | 0 | | | | | | |
|  |  | | | | | | |
|  |  | | | | | | |
| **Figure specifications (please complete one row per figure)^5^**  ***Figure no.*** | Colour  ***(yes/no)*** | Greyscale  ***(yes/no)*** | Black and white  ***(yes/no)*** | Single column (80mm)  ***(yes/no)*** | Double column (180mm)  ***(yes/no)*** | Size of figure at full scale  (mm x mm)  ***(insert details)*** | Smallest font size used in the figure at full scale (minimum 6pt)  ***(insert***  ***details)*** |
| 1 | yes |  |  |  |  | 160 x 117.4 | 6 |
| 2 | yes |  |  |  |  | 180 x 140.6 | 6 |
| 3 | yes |  |  |  |  | 125.8 x 159.2 | 6 |
| 4 | yes |  |  |  |  | 142.7 x 159.2 | 6 |
| 5 | yes |  |  |  |  | 206.6 x 104.4 | 7 |
|  |  |  |  |  |  |  |  |
|  |  |  |  |  |  |  |  |

**^1^** The maximum character count allowed is 50,000 (incl. spaces) for Primary Research Papers and Reviews, 10,000 for Short Takes.

**^2^** Summary should not exceed 250 words.

**^3^** Primary Research Papers can contain a maximum of two tables. If more are needed they should replace some of the Figures or can be placed in the Supporting Information.

**^4^** A maximum of 45 references is allowed for Primary Research Papers and 20 references for Short Takes.

**^5^** A Primary Research Paper may contain up to 6 figures and a Short Take up to 2 figures. Authors are encouraged to provide figures in the size they are to appear in the journal and at the specifications given.
